# [Bis(2,6-diiso­propyl­phen­yl) phosphato-κ*O*]pentakis­(methanol-κ*O*)manganese bis­(2,6-diiso­propyl­phen­yl) phosphate methanol tris­olvate

**DOI:** 10.1107/S2056989018012859

**Published:** 2018-09-14

**Authors:** Mikhail E. Minyaev, Alexander N. Tavtorkin, Sof’ya A. Korchagina, Ilya E. Nifant’ev, Andrei V. Churakov, Artem O. Dmitrienko, Konstantin A. Lyssenko

**Affiliations:** a A.V. Topchiev Institute of Petrochemical Synthesis, Russian Academy of Sciences, 29 Leninsky Prospect, 119991, Moscow, Russian Federation; b Chemistry Department, M.V. Lomonosov Moscow State University, 1 Leninskie Gory Str., Building 3, Moscow 119991, Russian Federation; c N.S. Kurnakov Institute of General and Inorganic Chemistry, Russian Academy of Sciences, 31 Leninsky Prospect, Moscow 119991, Russian Federation; d A.N. Nesmeyanov Institute of Organoelement Compounds, Russian Academy of Sciences, 28 Vavilova Str., Moscow, 119991, Russian Federation

**Keywords:** manganese, organophosphate, hydrogen bonding, coordination compound, polydi­methyl­siloxane, thermal oxidation, crystal structure

## Abstract

The title compound, [Mn(C_24_H_34_O_4_P)(CH_3_OH)_5_](C_24_H_34_O_4_P)·3CH_3_OH, was formed in the reaction between a hydrate of a manganese(II) salt [either Mn(NO_3_)_2_(H_2_O)_6_ or MnCl_2_(H_2_O)_4_] with a methanol solvate of lithium bis­(2,6-diiso­propyl­phen­yl) phosphate, {Li[OOP(O-2,6-^*i*^Pr_2_C_6_H_3_)_2_]·(CH_4_O)_3_}·CH_4_O, in methanol. The structure has monoclinic (*Cc*) symmetry at 150 K. The complex consists of an [Mn{OOP(O-2,6-^*i*^Pr_2_C_6_H_3_)_2_}(CH_3_OH)_5_]^+^ cation, an [OOP(O-2,6-^*i*^Pr_2_C_6_H_3_)_2_]^−^ anion and three non-coordinating methanol mol­ecules. The anion demonstrates disorder of an isopropyl group [occupancy ratio is 0.57 (4):0.43 (4)]. The di­aryl­phosphate ligand in the cation exhibits a κ^1^ *O* terminal coordination mode. The Mn atom is in a nearly unperturbed octa­hedral environment. The [Mn{OOP(O-2,6-^*i*^Pr_2_C_6_H_3_)_2_}(CH_3_OH)_5_]^+^ cation exhibits one intra­molecular O—H⋯O bond, and is coordinated *via* two inter­molecular O—H⋯O hydrogen bonds to the [OOP(O-2,6-^*i*^Pr_2_C_6_H_3_)_2_]^−^ anion. The cations, anions and non-coordinating methanol mol­ecules are linked into infinite chains along the *c*-axis direction *via* 0—H⋯O hydrogen bonding. The complex is of inter­est as a possible inhibitor for the thermal decomposition of polydi­methyl­siloxane. The crystal studied was refined as an inversion twin with a domain ratio of 0.47 (3):0.53 (3).

## Abstract 2

The crystal structure of the complex [Mn{OOP(O-2,6-^*i*^Pr_2_C_6_H_3_)_2_}(CH_3_OH)_5_]^+^[OOP(O-2,6-^*i*^Pr_2_C_6_H_3_)_2_]^−^·(CH_3_OH)_3_ exhibits O—H⋯O bonds between the cations, anions and non-coordinating methanol mol­ecules, forming infinite one-dimensional associates. The complex demonstrates inhibition of thermal oxidation of polydi­methyl­siloxane.

## Chemical context   

Polydi­methyl­siloxane (PDMS) liquids are widely applied in many devices as shock-absorbing, hydraulic and damping liquids, as bases for greases and as heat-transfer agents for many industrial processes carried out at elevated temperatures. Various lipophilic derivatives of metals with variable valency, such as Mn, Fe, Ni, Ce, *etc*., are used for the inhibition of thermo-oxidative decomposition of polyorganosiloxane heat carriers (Swihart & Jones, 1985 ▸; Nielsen, 1961 ▸; Halm, 1980 ▸; Kobzova *et al.*, 1966 ▸; Kishimoto *et al.*, 1976 ▸; Rozanova *et al.*, 1995 ▸; Minyaev *et al.*, 2018*a* ▸) in order to increase their operating time and temperature (usually up to *ca* 550 K). As manganese-based inhibitors, cymantrene and its derivatives have shown promising results (Sobolevskiy *et al.*, 1970 ▸). However, these Mn compounds are not available on an industrial scale. Easily accessible disubstituted organophosphate ligands are usually regarded as being lipophilic. For example, rare-earth complexes with such disubstituted organophosphate ligands are highly soluble in hydro­carbon media (Nifant’ev *et al.*, 2013 ▸, 2014 ▸). Therefore, the obtained manganese derivative with the organophosphate ligand might be a readily available alternative to cymantrene and to its derivatives.

Herein we report on the crystal structure of the Mn organo­phosphate complex [Mn{OOP(O-2,6-^*i*^Pr_2_C_6_H_3_)_2_}(CH_3_OH)_5_]^+^[OOP(O-2,6-^*i*^Pr_2_C_6_H_3_)_2_]^−^·3CH_3_OH, which contains a lipophilic diaryl-substituted organophosphate ligand, and on its properties regarding inhibition of the thermal oxidation of polydi­methyl­siloxane.
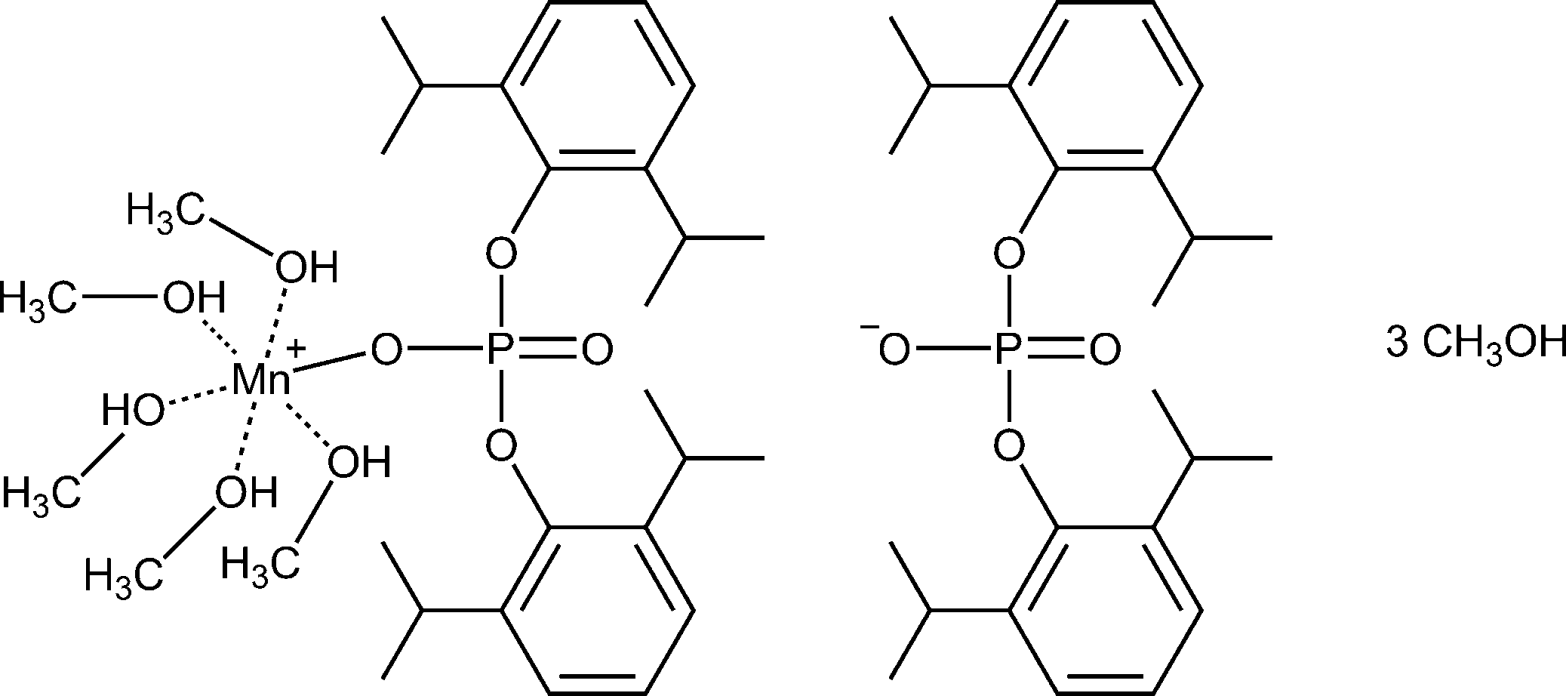


**Figure 1:**
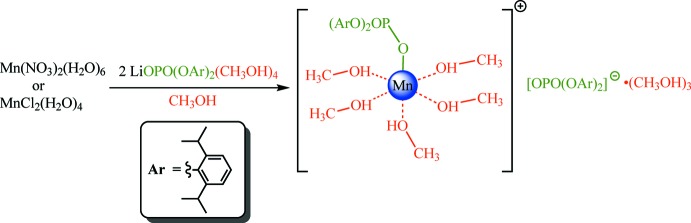
Synthesis of [Mn{(2,6-^*i*^Pr_2_C_6_H_3_-O)_2_PO_2_}(CH_3_OH)_5_][(2,6-^*i*^Pr_2_C_6_H_3_-O)_2_PO_2_]·(CH_3_OH)_3_.

The title compound can be synthesized (Fig. 1 ▸) by the reaction of either manganese(II) nitrate hexa­hydrate, Mn(NO_3_)_2_(H_2_O)_6_, or manganese(II) chloride tetra­hydrate, MnCl_2_(H_2_O)_4_, with lithium bis­(2,6-diiso­propyl­phen­yl) phosphate methanol solvate, {Li[OOP(O-2,6-^*i*^Pr_2_C_6_H_3_)_2_](CH_3_OH)_3_}·CH_3_OH (for its structure, see Minyaev *et al.*, 2015 ▸). Performing the reaction in a methanol medium provided the ionic complex instead of the expected neutral complex.

## Analysis of thermal decomposition inhibition properties   

We tested the title Mn compound as a possible inhibitor for the thermal decomposition of the heat-transfer agent PDMS in air at a temperature of 573 K, and compared the obtained results with control experiments and with experiments, where the Ce complex [Ce{O_2_P(O-2,6-^*i*^Pr_2_C_6_H_3_)_2_}_2_(CH_3_OH)_5_]·CH_3_OH bearing the same ligand was used (Minyaev *et al.*, 2018*a* ▸). All experiments were carried out under the same conditions (Table 1 ▸).

The results indicate that the manganese derivative inhibits the thermal decomposition of the silicone heat carrier, although to a much lesser extent than the cerium derivative at the same loads (each 0.1% by mass, entries 2 and 4). Moreover, the PDMS liquid containing 0.1% of the Mn complex became solidified at the end of the experiment. However, with an increase of the manganese derivative load of up to 0.5% (entry 3), the PDMS decomposition decreases to the level displayed by the cerium additive at 0.1%. Thus, the lipophilic manganese derivative may be used as an accessible alternative to cerium and organometallic manganese derivatives.

## Structural commentary   

**Figure 2:**
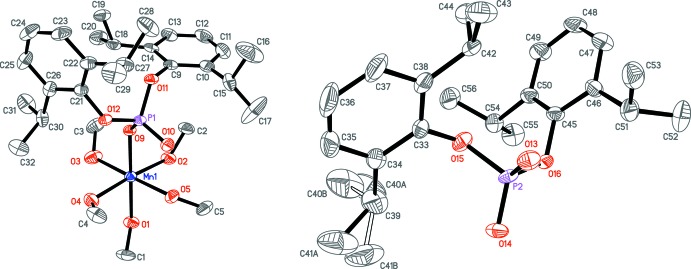
The structures of the [Mn{OOP(O-2,6-^*i*^Pr_2_C_6_H_3_)_2_}(CH_3_OH)_5_]^+^ cation (left) and [OOP(O-2,6-^*i*^Pr_2_C_6_H_3_)_2_]^−^ anion (right). Displacement ellipsoids are drawn at the 50% probability level. Hydrogen atoms have been omitted for clarity.

**Figure 3:**
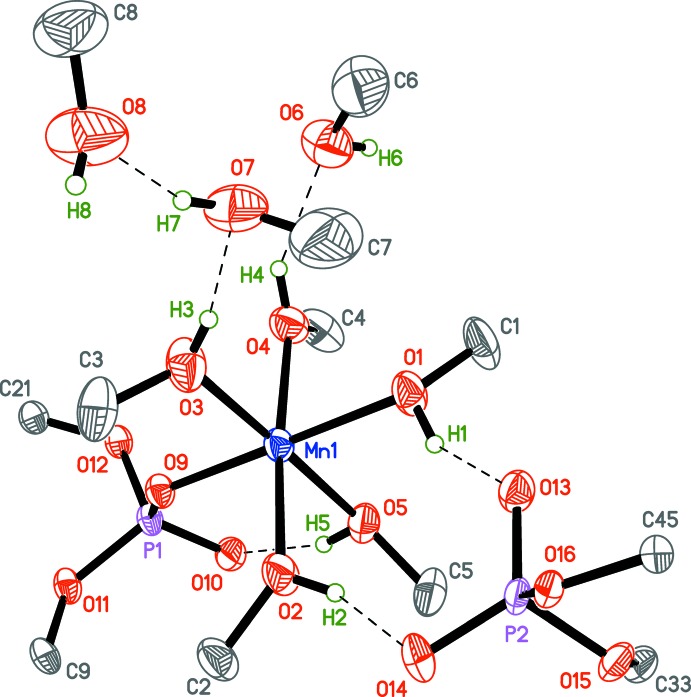
The asymmetric unit and hydrogen bonding within it. Displacement ellipsoids are drawn at the 50% probability level. Only hy­droxy H atoms and only C_*ipso*_ atoms (C9, C21, C33 and C45) of aryl groups are shown for clarity.

The mol­ecular components of the title compound comprise an [Mn{O_2_P(O-2,6-^*i*^Pr_2_C_6_H_3_)_2_}(CH_3_OH)_5_]^+^ cation (Fig. 2 ▸, left), an [O_2_P(O-2,6-^*i*^Pr_2_C_6_H_3_)_2_]^−^ anion (Fig. 2 ▸, right) and three non-coordinating methanol mol­ecules (Fig. 3 ▸). The bis­(2,6-diiso­propyl­phen­yl)phosphate ligand in the cation exhibits a κ^1^ *O* terminal coordination mode. The Mn^2+^ cation is also coordinated by five methanol mol­ecules, providing a nearly unperturbed octa­hedral environment. The Mn—O_methanol_ bond distances range from 2.146 (3) to 2.236 (4) Å, whereas the Mn—O_phosphate_ bond length is shorter, with a value of 2.116 (3) Å (Table 2 ▸). The shortest Mn—O_Methanol_ bond (Mn—O1) is at the *trans*-position to the Mn—O_phosphate_ bond. The O—Mn—O bond angles between two neighboring ligands (at the *cis*-positions) are very close to 90° and vary between 86.88 (14)° [O1—Mn—O4] and 93.86 (13)° [O2—Mn—O9]. The O—Mn—O angles between *trans*-ligands range from 175.26 (14)° [O2—Mn—O4] to 178.62 (16)° [O3—Mn—O5].

The O—C_*ipso*_ bond distances [which range from 1.403 (5) Å for O12–C21 to 1.409 (5) Å for O16—C45] correspond to those of a slightly shortened regular single O—C bond (1.43 Å), indicating no significant charge redistribution between the PO_4_ and aryl fragments. Both phospho­rous atoms adopt distorted tetra­hedral environments. The value of the P—O_Mn_ distance [P1—O9 = 1.503 (3) Å] is very close to the P—O distances for O atoms that are not connected to any other non-H atoms in both phosphate groups [1.488 (3)–1.496 (4) Å for the P1—O10, P2—O13 and P2—O14 bonds; see Table 2 ▸]. This indicates a mainly ionic character of the Mn—phosphate bond. The P—O_C_ bond lengths are considerably higher [1.597 (3)–1.607 (3) Å]. Regardless of aryl steric hindrance, the O_C_—P—O_C_ bond angles are the smallest [100.3 (2)° for O11—P1—O12 and 99.3 (2)° for O15—P2—O16] among all of the O—P—O angles, which range from 105.8 (2)° for O10—P1—O12 to 117.1 (2)° for O13—P2—O14.

All of these facts point not only to an approximately equal negative charge redistribution on atoms O9, O10 and O13, O14, but also to more pronounced double-bond character for the corresponding P—O bonds compared to the P—O_C_ bonds. These results are in good agreement with data obtained for rare-earth phosphates bearing the same ligand: [*Ln*{O_2_P(O-2,6-^*i*^Pr_2_C_6_H_3_)_2_}_2_Cl(CH_3_OH)_4_]·2CH_3_OH (*Ln* = Nd, Lu, Y; Minyaev *et al.*, 2017 ▸), [*Ln*{O_2_P(O-2,6-^*i*^Pr_2_C_6_H_3_)_2_}_3_(CH_3_OH)_5_]·CH_3_OH (*Ln* = La, Ce, Nd; Minyaev *et al.*, 2018*a* ▸), {La_2_[(2,6-^*i*^Pr_2_C_6_H_3_-O)_2_POO]_5_(H_2_O)_2_(OH)}·2(hexa­ne) and {Nd_2_[(2,6-^*i*^Pr_2_C_6_H_3_-O)_2_POO]_4_(H_2_O)_4_(OH)}^+^[(2,6-^*i*^Pr_2_C_6_H_3_-O)_2_POO]^−^·2(hepta­ne) (Minyaev *et al.*, 2018*b* ▸).

## Supra­molecular features   

**Figure 4:**
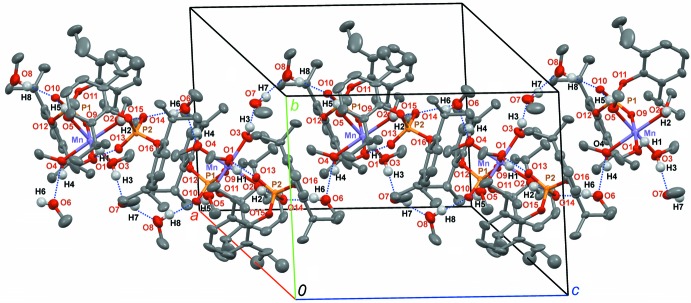
An infinite one-dimensional supramolecular chain {[Mn{OOP(O-2,6-^*i*^Pr_2_C_6_H_3_)_2_}(CH_3_OH)_5_]^+^[OOP(O-2,6-^*i*^Pr_2_C_6_H_3_)_2_]^−^·3CH_3_OH_3_}_∞_ formed by O—H⋯O bonds (blue dashed lines). Displacement ellipsoids are drawn at the 50% probability level. Disorder is not shown.

The [Mn{O_2_P(O-2,6-^*i*^Pr_2_C_6_H_3_)_2_}(CH_3_OH)_5_]^+^ cation exhibits one intra­molecular hydrogen bond (O5—H5⋯O10, Table 3 ▸). The [OOP(O-2,6-^*i*^Pr_2_C_6_H_3_)_2_]^−^ anion and the cation are connected *via* two hydrogen bonds: O1—H1⋯O13 and O2—H2⋯O14. The cation is also connected to the non-coordin­ating methanol mol­ecules *via* O3—H3⋯O7 and O4—-H4⋯O6 hydrogen bonds, and further linked to the third mol­ecule by the O7—H7⋯O8 hydrogen bond, forming the supramolecular moiety shown in Fig. 3 ▸. These moieties are linked by O6—H6⋯O14^i^ and O8—H8⋯O10^ii^ bonds [symmetry codes: (i) *x*, −*y* + 1, *z* − 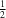; (ii) *x*, −*y* + 1, *z* + 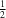; see Table 3 ▸], forming infinite chains along the *c*-axis direction (Fig. 4 ▸).

The presence of two separate ions in the crystal lattice can be explained by the relatively large solvation energy obtained from the formation of many O—H⋯O bonds within a one-dimensional hydrogen-bond network. This might be one of the driving forces for crystal formation.

## Database survey   

The crystal structures of manganese complexes with various di-substituted organophosphate ligands have not yet been studied well. Thus, the number of structures in the Cambridge Structural Database (CSD version 5.38, latest update May 2017; Groom *et al.*, 2016 ▸) is limited to 20 (after the exclusion of duplicated structures). These comprise: one mononuclear complex (MOKCEU; Murugavel & Sathiyendiran, 2001 ▸); four binuclear complexes [DAVFEM (Shiraishi *et al.*, 2005 ▸), ENIMUJ (Yashiro *et al.*, 2003 ▸), YIWYUA and YIWZAH (Pothi­raja *et al.*, 2014 ▸)]; three tetra­nuclear complexes (YOSPIH, YOSPON and YOSPUT; Van Allsburg *et al.*, 2015 ▸); two trinuclear heterometallic complexes [ENEHAI (Nakajima *et al.*, 2016 ▸) and RITKIO (Dean *et al.*, 1997 ▸)]; two dodeca­nuclear complexes [DAGJEB/DAGJEB01 (Bian *et al.*, 2004 ▸; Kuroda-Sowa *et al.*, 2005 ▸) and XUBXOH/XUBXOH01 (Kuroda-Sowa *et al.*, 2002 ▸, 2005 ▸)]; eight coordination polymers [KOZZAC and KOZZUW (Rajakannu *et al.*, 2015 ▸), LULGEE (Sathiyendiran & Murugavel, 2002 ▸), ODEWOK (Rafizadeh *et al.*, 2007 ▸), SAMNEA/SAMNEA01 (Pothiraja *et al.*, 2004 ▸, 2005 ▸), TEKQOR and TEKQUX (Dey *et al.*, 2013 ▸) and WENSUE (Rafizadeh *et al.*, 2006 ▸)]. All of the above are heteroleptic complexes containing the following di-substituted organophosphate ligands: PO_2_(OPh)_2_, PO_2_(OC_6_H_4_-4-NO_2_)_2_, PO_2_(OMe)_2_, PO_2_(O^*t*^Bu)_2_ and PO_2_(OCMe_2_CMe_2_O). The ligands mainly display a μ_2_-κ^1^ *O*:κ^1^ *O*′ bridging coordination mode, and occasionally a κ^1^ *O* terminal mode. The Mn complexes, especially mononuclear ones, with other disubstituted organophosphate anions are yet to be synthesized. It is worth mentioning that the tile complex is mononuclear, incorporates a novel organo­phosphate ligand, and is the first Mn–phosphate complex with a phosphate anion separated from the Mn complex cation in the crystal lattice.

## Synthesis and crystallization   

### General experimental remarks   

The synthesis of the title complex was carried out under an argon atmosphere. Lithium bis­(2,6-diiso­propyl­phen­yl) phosphate methanol tetra­solvate, [Li{OOP(O-2,6-^*i*^Pr_2_C_6_H_3_)_2_}(CH_3_OH)_3_]·CH_3_OH, was synthesized according to the literature procedure (Minyaev *et al.*, 2015 ▸). C/H elemental analysis was performed with a Perkin–Elmer 2400 Series II elemental analyzer. Methanol was distilled over a Ca/Mg alloy and stored over mol­ecular sieves (4 Å). Polydi­methyl­siloxane (PDMS-50, viscosity 50 mm^2^ s^−1^) was used as purchased (Sofex–Silicone). XRF studies were performed with an ARL ADVANTIX instrument. Powder patterns (supplementary Figs. S1–S5) were recorded on a Bruker D8 Advance Vario diffractometer, using Cu *K*α_1_ radiation [Ge(111) monochromator] and a LynxEye 1D position-sensitive detector in transmission mode at room temperature. The 2θ range was 2–90° with a 0.01° step for all samples. The Rietveld analysis was carried out with *Topas* software (Bruker, 2015 ▸).

### Synthesis and crystallization of the complex   

A solution of Mn(NO_3_)_2_(H_2_O)_6_ (159 mg, 0.55 mmol) in 5 ml of methanol was carefully added to a solution of [Li{OOP(O-2,6-^*i*^Pr_2_C_6_H_3_)_2_}(CH_3_OH)_3_]·CH_3_OH (580 mg, 1.05 mmol) in 5 ml of methanol at room temperature. The mixture was stirred for 10 s. Crystals started to precipitate out after 20 min.. The following day, some crystals were taken from the mother liquor for X-ray studies. The remaining crystals were filtered off, washed with methanol (2 × 10 ml) and dried briefly under dynamic vacuum [yield 485 mg (0.42 mmol, 81%) as colourless prismatic crystals. Analysis found (calculated for C_56_H_100_MnO_16_P_2_): C 58.75 (58.68), H 8.72% (8.79%). The same com­pound was prepared in 80% yield from MnCl_2_(H_2_O)_4_ under similar reaction conditions. The crystal shapes varied from needles to blocks, depending on the synthesis and crystal growth conditions. The formed high-spin Mn complex cannot be studied by NMR techniques because of its paramagnetic behaviour.

### Thermal oxidation of polydi­methyl­siloxane   

A mixture (2.000 g) of the Mn complex (either 2 mg or 10 mg) and PDMS was placed in a glass beaker. No additive was used in the control experiments. The beaker was placed into a muffle furnace with a preset temperature of 573 K. The beaker was periodically taken out from the furnace and weighed to determine the weight loss.

## Refinement   

Crystal data, data collection and structure refinement details are summarized in Table 4 ▸. The positions of most hydrogen atoms were found from the difference electron-density map, but they were positioned geometrically (C—H = 0.95 Å for aromatic, 0.98 Å for methyl and 0.99 Å for methyl­ene H atoms) and refined as riding atoms with relative isotropic displacement parameters *U*_iso_(H) = 1.5U_eq_(C) for methyl H atoms and 1.2*U*_eq_(C) otherwise. The positions of the hy­droxy H atoms were refined with restrained O—H distances of 0.85 (2) Å with *U*_iso_(H)= 1.2*U*_eq_(O). A rotating group model was applied for methyl groups. Two reflections (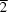 0 0 and 2 0 0) were affected by the beam stop, and were therefore omitted from the refinement. Two reflections (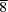 2 10 and 4 0 4) were also omitted from the final cycles of the refinement as their (*I*_obs_ − *I*_calcd_)/σ(*w*) values were over 10.

One of the isopropyl groups is disordered over two sets of sites with an occupancy ratio of 0.57 (4):0.43 (4) for atoms C40*A*/C41*A* and C40*B*/C41*B*, respectively. Four HC—CH_3_ distances in the disordered fragment were restrained to be equal within an estimated standard deviation of 0.01 Å. Similarity restraints for thermal displacement ellipsoids were also applied. The crystal studied was refined as an inversion twin with a domain ratio of 0.47 (3):0.53 (3).

The final crystallographic model exhibits some problems, including two relatively high remaining *Q* peaks of residual electron density, which could not be reasonably handled, and a rather high Δρ_max_/Δρ_min_ ratio.

The problems might have been caused by (1) incomplete substitution of NO_3_^−^ in crystals initially made from Mn(NO_3_)_2_(H_2_O)_6_, (2) some content of other metal impurities, (3) crystal decomposition during data collection, (4) twinning or (5) disorder. Several attempts to prepare crystal batches were made, starting from Mn(NO_3_)_2_(H_2_O)_6_ and from MnCl_2_(H_2_O)_4_ by varying the crystal-growth conditions slightly. Several attempts to reestablish the crystal structure were made using different diffractometers and software (see Table S1 in the supporting information for details). Crystallographic models of the studied crystals demonstrated the same problems regardless of differences in the preparation and the instrument used. Modelling disorder and applying various twinning laws (using *CELL_NOW*) were unsuccessful. The X-ray fluorescence (XRF) analysis demonstrated the presence of only the elements P and Mn and the absence of a noticeable qu­antity of any other heavy element (heavier than Ne). Several C/H analyses undertaken immediately after the crystal preparation showed very similar results that were nearly identical to calculated values.

Inter­esting results were obtained by using the powder X-ray diffraction (pXRD) method (see the supporting information). After several days without being in the solvent, the sample became non-single-phased. Moreover, the sample demonstrated dramatic changes in its phase composition during the pXRD measurements (see Figs. S2–S5). Such a phase change might be attributed to the facile loss of non-coordinating methanol mol­ecules.

Therefore, the inherent problems of the presented crystallographic model can only be the result of slow crystal decomposition during the X-ray measurements or/and, more likely, from some subtle unrevealed twinning.

## Supplementary Material

Crystal structure: contains datablock(s) I. DOI: 10.1107/S2056989018012859/pj2055sup1.cif

Supporting information file. DOI: 10.1107/S2056989018012859/pj2055Isup3.cdx

Structure factors: contains datablock(s) I. DOI: 10.1107/S2056989018012859/pj2055Isup4.hkl

Powder XRD data and single crystal X-ray diffraction studies. DOI: 10.1107/S2056989018012859/pj2055sup5.pdf

CCDC reference: 1867164

Additional supporting information:  crystallographic information; 3D view; checkCIF report

## Figures and Tables

**Table 1:** Weight loss (%) *versus* time and gel time (h) in the thermal destruction of PDMS The starting mass of PDMS-50 was 2.000 g. The thermal destruction experiments were carried out at *T* = 573 K. The Mn complex is [Mn{O_2_P(O-2,6-^*i*^Pr_2_C_6_H_3_)_2_}(CH_3_OH)_5_]^+^[O_2_P(O-2,6-^*i*^Pr_2_C_6_H_3_)_2_]^−^·3CH_3_OH and the Ce complex is [Ce{O_2_P(O-2,6-^*i*^Pr_2_C_6_H_3_)_2_}_2_(CH_3_OH)_5_]·CH_3_OH.

Entry	Additive	Weight loss					Gel time^*a*^
		1 h	2 h	3 h	5 h	9 h	
1	None (control)	1.5%	3.5%	5.5%	9%	13.5%	5 h
2	0.1% Mn	1%	2%	3%	6%	8.5%	9 h
3	0.5% Mn	1%	2%	2.5%	3.5%	6%	–^*b*^
4	0.1% Ce	1%	1.5%	2%	3%	4.5%	–^*b*^

Notes: (*a*) After this time, the PDMS liquid becomes fully solidified. (*b*) No precipitate, low viscosity, clear liquid at the end of the experiment (9 h).

**Table 2:** Selected bond lengths (Å)

Mn1—O1	2.146 (3)	P1—O10	1.488 (3)
Mn1—O2	2.236 (4)	P1—O11	1.600 (3)
Mn1—O3	2.158 (4)	P1—O12	1.597 (3)
Mn1—O4	2.213 (4)	P2—O13	1.496 (4)
Mn1—O5	2.220 (4)	P2—O14	1.488 (3)
Mn1—O9	2.116 (3)	P2—O15	1.607 (3)
P1—O9	1.503 (3)	P2—O16	1.600 (3)

**Table 3:** Hydrogen-bond geometry (Å, °)

*D*—H⋯*A*	*D*—H	H⋯*A*	*D*⋯*A*	*D*—H⋯*A*
O1—H1⋯O13	0.85	1.79	2.537 (5)	145
O2—H2⋯O14	0.82	1.99	2.724 (5)	148
O3—H3⋯O7	0.86	1.79	2.644 (6)	170
O4—H4⋯O6	0.85	1.95	2.700 (7)	147
O5—H5⋯O10	0.85	1.84	2.661 (5)	163
O6—H6⋯O14^i^	0.86	1.89	2.708 (6)	157
O7—H7⋯O8	0.85	1.88	2.697 (8)	159
O8—H8⋯O10^ii^	0.85	1.86	2.708 (7)	174

Symmetry codes: (i) 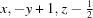; (ii) 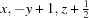.

**Table 4:** Experimental details

Crystal data	
Chemical formula	[Mn(C_24_H_34_O_4_P)(CH_4_O)_5_](C_24_H_34_O_4_P)·3CH_4_O
*M*_r_	1146.23
Crystal system, space group	Monoclinic, *C* *c*
Temperature (K)	150
*a*, *b*, *c* (Å)	31.872 (6), 12.640 (2), 16.881 (3)
β (°)	109.990 (2)
*V* (Å^3^)	6391 (2)
*Z*	4
Radiation type	Mo *K*α
μ (mm^−1^)	0.32
Crystal size (mm)	0.40 × 0.40 × 0.25

Data collection	
Diffractometer	Bruker SMART APEXII
Absorption correction	Multi-scan (*SADABS*; Bruker, 2008 ▸)
*T*_min_, *T*_max_	0.724, 0.923
No. of measured, independent and observed [*I* > 2σ(*I*)] reflections	36871, 18839, 16119
*R*_int_	0.031
(sin θ/λ)_max_ (Å^−1^)	0.714

Refinement	
*R*[*F* ^2^ > 2σ(*F* ^2^)], *wR*(*F* ^2^), *S*	0.072, 0.197, 1.07
No. of reflections	18839
No. of parameters	730
No. of restraints	52
H-atom treatment	H atoms treated by a mixture of independent and constrained refinement
Δρ_max_, Δρ_min_ (e Å^−3^)	3.11, −0.74
Absolute structure	Refined as an inversion twin
Absolute structure parameter	0.47 (2)

Computer programs: *APEX2* and *SAINT* (Bruker, 2008 ▸), *SHELXS2013* and *SHELXTL* (Sheldrick, 2008 ▸), *SHELXL2017* (Sheldrick, 2015 ▸), *publCIF* (Westrip, 2010 ▸) and *Mercury* (Macrae *et al.*,2006 ▸).

## supplementary crystallographic information

### Crystal data

**Table:**

[Mn(C_24_H_34_O_4_P)(CH_4_O)_5_](C_24_H_34_O_4_P)·3CH_4_O	*F*(000) = 2476
*M**_r_* = 1146.23	*D*_x_ = 1.191 Mg m^−^^3^
Monoclinic, *C**c*	Mo *K*α radiation, λ = 0.71073 Å
*a* = 31.872 (6) Å	Cell parameters from 9949 reflections
*b* = 12.640 (2) Å	θ = 2.3–30.5°
*c* = 16.881 (3) Å	µ = 0.32 mm^−^^1^
β = 109.990 (2)°	*T* = 150 K
*V* = 6391 (2) Å^3^	Prism, colourless
*Z* = 4	0.40 × 0.40 × 0.25 mm

### Data collection

**Table:**

Bruker SMART APEXII diffractometer	18839 independent reflections
Radiation source: fine-focus sealed tube	16119 reflections with *I* > 2σ(*I*)
Graphite monochromator	*R*_int_ = 0.031
ω scans	θ_max_ = 30.5°, θ_min_ = 2.0°
Absorption correction: multi-scan (SADABS; Bruker, 2008)	*h* = −45→45
*T*_min_ = 0.724, *T*_max_ = 0.923	*k* = −17→17
36871 measured reflections	*l* = −24→24

### Refinement

**Table:**

Refinement on *F*^2^	Secondary atom site location: difference Fourier map
Least-squares matrix: full	Hydrogen site location: mixed
*R*[*F*^2^ > 2σ(*F*^2^)] = 0.072	H atoms treated by a mixture of independent and constrained refinement
*wR*(*F*^2^) = 0.197	*w* = 1/[σ^2^(*F*_o_^2^) + (0.1189*P*)^2^ + 8.5165*P*] where *P* = (*F*_o_^2^ + 2*F*_c_^2^)/3
*S* = 1.07	(Δ/σ)_max_ = 0.002
18839 reflections	Δρ_max_ = 3.11 e Å^−^^3^
730 parameters	Δρ_min_ = −0.74 e Å^−^^3^
52 restraints	Absolute structure: Refined as an inversion twin
Primary atom site location: structure-invariant direct methods	Absolute structure parameter: 0.47 (2)

### Special details

**Table:**

Geometry. All esds (except the esd in the dihedral angle between two l.s. planes) are estimated using the full covariance matrix. The cell esds are taken into account individually in the estimation of esds in distances, angles and torsion angles; correlations between esds in cell parameters are only used when they are defined by crystal symmetry. An approximate (isotropic) treatment of cell esds is used for estimating esds involving l.s. planes.
Refinement. Refinement of F^2^ against ALL reflections. The weighted R-factor wR and goodness of fit S are based on F^2^, conventional R-factors R are based on F, with F set to zero for negative F^2^. The threshold expression of F^2^ > 2sigma(F^2^) is used only for calculating R-factors(gt) etc. and is not relevant to the choice of reflections for refinement. R-factors based on F^2^ are statistically about twice as large as those based on F, and R- factors based on ALL data will be even larger. Refined as a 2-component inversion twin.

### Fractional atomic coordinates and isotropic or equivalent isotropic displacement parameters (Å^2^)

**Table:**

	*x*	*y*	*z*	*U*_iso_*/*U*_eq_	Occ. (<1)
Mn1	0.51624 (2)	0.56670 (5)	0.44955 (4)	0.02200 (15)	
O1	0.45043 (11)	0.5084 (3)	0.4319 (2)	0.0317 (8)	
H1	0.4396 (4)	0.5541 (15)	0.4563 (8)	0.048*	
C1	0.41774 (19)	0.4808 (7)	0.3527 (4)	0.0469 (16)	
H1A	0.404356	0.412417	0.357752	0.070*	
H1B	0.432005	0.475751	0.309781	0.070*	
H1C	0.394447	0.535174	0.336204	0.070*	
O2	0.51222 (12)	0.6630 (3)	0.5578 (2)	0.0324 (8)	
H2	0.4873 (9)	0.6797 (7)	0.5578 (2)	0.049*	
C2	0.54381 (19)	0.7447 (5)	0.5988 (4)	0.0374 (12)	
H2A	0.528752	0.799432	0.620322	0.056*	
H2B	0.555995	0.776127	0.558241	0.056*	
H2C	0.568134	0.713868	0.645766	0.056*	
O3	0.54723 (14)	0.4397 (4)	0.5350 (3)	0.0419 (10)	
H3	0.5324 (5)	0.3856 (18)	0.5417 (4)	0.063*	
C3	0.5834 (2)	0.4602 (7)	0.6116 (4)	0.0498 (16)	
H3A	0.582758	0.408705	0.654509	0.075*	
H3B	0.580468	0.531835	0.631333	0.075*	
H3C	0.611784	0.454225	0.601316	0.075*	
O4	0.51420 (13)	0.4692 (3)	0.3394 (3)	0.0336 (8)	
H4	0.51595 (14)	0.402 (2)	0.3393 (3)	0.050*	
C4	0.5057 (2)	0.5088 (6)	0.2549 (4)	0.0444 (14)	
H4A	0.514894	0.455615	0.221773	0.067*	
H4B	0.522807	0.573981	0.257462	0.067*	
H4C	0.473811	0.523579	0.228182	0.067*	
O5	0.48595 (12)	0.6992 (3)	0.3625 (3)	0.0335 (8)	
H5	0.5086 (8)	0.7311 (11)	0.3584 (3)	0.050*	
C5	0.4553 (2)	0.7743 (6)	0.3730 (5)	0.0472 (15)	
H5A	0.442418	0.814878	0.320784	0.071*	
H5B	0.470828	0.822452	0.419211	0.071*	
H5C	0.431422	0.737556	0.386175	0.071*	
O6	0.48720 (19)	0.2694 (4)	0.2918 (3)	0.0506 (12)	
H6	0.4699 (6)	0.2887 (8)	0.2426 (17)	0.076*	
C6	0.4648 (3)	0.1958 (8)	0.3256 (7)	0.071 (3)	
H6A	0.455252	0.135345	0.287273	0.107*	
H6B	0.438547	0.229462	0.332550	0.107*	
H6C	0.484905	0.171313	0.380533	0.107*	
O7	0.5105 (2)	0.2605 (4)	0.5589 (4)	0.0547 (13)	
H7	0.5293 (7)	0.2376 (9)	0.6050 (16)	0.082*	
C7	0.4701 (4)	0.2874 (8)	0.5717 (10)	0.087 (3)	
H7A	0.447395	0.233779	0.545471	0.130*	
H7B	0.459763	0.356567	0.546185	0.130*	
H7C	0.475196	0.290601	0.632291	0.130*	
O8	0.5566 (3)	0.1457 (5)	0.6957 (4)	0.0733 (19)	
H8	0.5582 (3)	0.1795 (12)	0.7402 (15)	0.110*	
C8	0.5546 (4)	0.0388 (7)	0.7096 (6)	0.071 (3)	
H8A	0.582879	0.015402	0.751309	0.107*	
H8B	0.549415	0.000387	0.656664	0.107*	
H8C	0.530132	0.024446	0.730777	0.107*	
P1	0.59610 (3)	0.69092 (9)	0.40155 (6)	0.0186 (2)	
O9	0.58012 (10)	0.6249 (3)	0.4596 (2)	0.0206 (6)	
O10	0.56168 (12)	0.7604 (3)	0.3429 (2)	0.0274 (7)	
O11	0.63971 (10)	0.7556 (3)	0.4553 (2)	0.0229 (6)	
O12	0.61596 (10)	0.6216 (3)	0.3435 (2)	0.0207 (6)	
C9	0.64458 (15)	0.8004 (4)	0.5344 (3)	0.0250 (9)	
C10	0.62856 (17)	0.9022 (4)	0.5377 (4)	0.0325 (11)	
C11	0.6356 (2)	0.9445 (5)	0.6181 (5)	0.0437 (14)	
H11	0.625280	1.013925	0.622988	0.052*	
C12	0.6572 (2)	0.8867 (6)	0.6907 (4)	0.0446 (14)	
H12	0.660979	0.916022	0.744462	0.053*	
C13	0.67350 (19)	0.7859 (5)	0.6847 (3)	0.0361 (11)	
H13	0.688395	0.747236	0.734672	0.043*	
C14	0.66832 (16)	0.7409 (4)	0.6069 (3)	0.0255 (9)	
C15	0.6067 (2)	0.9678 (5)	0.4592 (4)	0.0424 (13)	
H15	0.602164	0.919607	0.409978	0.051*	
C16	0.6377 (4)	1.0557 (8)	0.4513 (7)	0.088 (4)	
H16A	0.663790	1.024608	0.441984	0.132*	
H16B	0.647472	1.097612	0.503294	0.132*	
H16C	0.621809	1.101472	0.403628	0.132*	
C17	0.5615 (4)	1.0072 (12)	0.4531 (8)	0.100 (4)	
H17A	0.539894	0.949104	0.436031	0.149*	
H17B	0.552412	1.064061	0.411173	0.149*	
H17C	0.562474	1.034393	0.508085	0.149*	
C18	0.68866 (16)	0.6365 (4)	0.6000 (3)	0.0288 (10)	
H18	0.666495	0.596753	0.552851	0.035*	
C19	0.73069 (17)	0.6506 (5)	0.5770 (4)	0.0349 (11)	
H19A	0.723064	0.687691	0.522881	0.052*	
H19B	0.743288	0.581107	0.572412	0.052*	
H19C	0.752679	0.692168	0.620832	0.052*	
C20	0.6993 (2)	0.5684 (6)	0.6793 (4)	0.0432 (14)	
H20A	0.672884	0.564327	0.696477	0.065*	
H20B	0.724067	0.600105	0.724787	0.065*	
H20C	0.707638	0.497050	0.667457	0.065*	
C21	0.65996 (15)	0.5850 (4)	0.3640 (3)	0.0229 (8)	
C22	0.69048 (16)	0.6485 (4)	0.3443 (3)	0.0270 (9)	
C23	0.73334 (17)	0.6070 (6)	0.3618 (4)	0.0380 (12)	
H23	0.755256	0.648890	0.350039	0.046*	
C24	0.74489 (19)	0.5081 (6)	0.3953 (4)	0.0432 (14)	
H24	0.774325	0.482028	0.406709	0.052*	
C25	0.7130 (2)	0.4466 (5)	0.4124 (4)	0.0394 (13)	
H25	0.720828	0.377781	0.435336	0.047*	
C26	0.66984 (17)	0.4836 (4)	0.3968 (3)	0.0279 (9)	
C27	0.67872 (18)	0.7571 (5)	0.3055 (3)	0.0321 (10)	
H27	0.647754	0.774791	0.303253	0.038*	
C28	0.7104 (3)	0.8409 (6)	0.3582 (5)	0.0548 (18)	
H28A	0.712653	0.833643	0.417308	0.082*	
H28B	0.698883	0.911374	0.337689	0.082*	
H28C	0.739952	0.831668	0.353447	0.082*	
C29	0.6796 (3)	0.7569 (6)	0.2156 (4)	0.0478 (15)	
H29A	0.659885	0.700978	0.183023	0.072*	
H29B	0.710112	0.744111	0.216873	0.072*	
H29C	0.669238	0.825632	0.189213	0.072*	
C30	0.6346 (2)	0.4124 (4)	0.4107 (3)	0.0316 (10)	
H30	0.610950	0.458936	0.418554	0.038*	
C31	0.6530 (3)	0.3440 (6)	0.4892 (4)	0.0498 (16)	
H31A	0.666722	0.389301	0.538407	0.075*	
H31B	0.675575	0.295508	0.482408	0.075*	
H31C	0.628722	0.303066	0.497385	0.075*	
C32	0.6127 (2)	0.3442 (6)	0.3331 (4)	0.0435 (14)	
H32A	0.587165	0.306581	0.339576	0.065*	
H32B	0.634398	0.292885	0.326952	0.065*	
H32C	0.602394	0.389357	0.282955	0.065*	
P2	0.40310 (3)	0.65637 (9)	0.57832 (6)	0.0196 (2)	
O13	0.40161 (12)	0.5762 (3)	0.5122 (2)	0.0271 (7)	
O14	0.44699 (11)	0.7068 (3)	0.6233 (2)	0.0277 (7)	
O15	0.36432 (11)	0.7424 (3)	0.5400 (2)	0.0268 (7)	
O16	0.38666 (10)	0.6071 (3)	0.6500 (2)	0.0207 (6)	
C33	0.35548 (16)	0.7897 (4)	0.4612 (3)	0.0262 (9)	
C34	0.37518 (19)	0.8881 (4)	0.4574 (4)	0.0374 (12)	
C35	0.3646 (2)	0.9357 (5)	0.3782 (5)	0.0500 (17)	
H35	0.377233	1.002428	0.373348	0.060*	
C36	0.3366 (3)	0.8874 (6)	0.3081 (5)	0.058 (2)	
H36	0.330423	0.920113	0.254656	0.070*	
C37	0.3166 (3)	0.7909 (6)	0.3135 (4)	0.0518 (17)	
H37	0.296772	0.759342	0.263690	0.062*	
C38	0.32544 (18)	0.7394 (4)	0.3912 (3)	0.0334 (11)	
C39	0.4047 (2)	0.9445 (5)	0.5350 (6)	0.058 (2)	
H39A	0.422484	0.887536	0.572793	0.070*	0.57 (4)
H39B	0.407375	0.893641	0.581821	0.070*	0.43 (4)
C40A	0.3824 (9)	1.002 (2)	0.586 (2)	0.082 (6)	0.57 (4)
H40A	0.404196	1.018163	0.641604	0.123*	0.57 (4)
H40B	0.358483	0.958016	0.592595	0.123*	0.57 (4)
H40C	0.369636	1.068167	0.557448	0.123*	0.57 (4)
C41A	0.4384 (8)	1.0114 (19)	0.5135 (17)	0.080 (6)	0.57 (4)
H41A	0.460891	0.965505	0.503487	0.121*	0.57 (4)
H41B	0.452990	1.059465	0.560364	0.121*	0.57 (4)
H41C	0.423300	1.052677	0.462546	0.121*	0.57 (4)
C40B	0.3777 (10)	1.034 (2)	0.551 (3)	0.080 (8)	0.43 (4)
H40D	0.350731	1.006181	0.558900	0.121*	0.43 (4)
H40E	0.369220	1.082706	0.502804	0.121*	0.43 (4)
H40F	0.395541	1.072513	0.601940	0.121*	0.43 (4)
C41B	0.4512 (6)	0.975 (3)	0.548 (3)	0.085 (8)	0.43 (4)
H41D	0.469580	0.910790	0.555035	0.128*	0.43 (4)
H41E	0.462694	1.019171	0.598049	0.128*	0.43 (4)
H41F	0.452287	1.014068	0.498424	0.128*	0.43 (4)
C42	0.30163 (18)	0.6380 (4)	0.3977 (4)	0.0359 (12)	
H42	0.321929	0.595733	0.445600	0.043*	
C43	0.2902 (2)	0.5700 (5)	0.3168 (5)	0.0513 (18)	
H43A	0.276166	0.503786	0.324824	0.077*	
H43B	0.317642	0.554119	0.305213	0.077*	
H43C	0.269586	0.609055	0.269214	0.077*	
C44	0.2597 (2)	0.6629 (5)	0.4174 (4)	0.0413 (13)	
H44A	0.267737	0.701560	0.470804	0.062*	
H44B	0.244612	0.596832	0.421899	0.062*	
H44C	0.239707	0.706440	0.372085	0.062*	
C45	0.34258 (14)	0.5721 (4)	0.6342 (3)	0.0233 (8)	
C46	0.33076 (16)	0.4681 (4)	0.6054 (3)	0.0279 (9)	
C47	0.28661 (19)	0.4370 (5)	0.5914 (4)	0.0365 (12)	
H47	0.277268	0.368134	0.570252	0.044*	
C48	0.25617 (17)	0.5045 (5)	0.6078 (4)	0.0390 (13)	
H48	0.226544	0.481295	0.598748	0.047*	
C49	0.26909 (16)	0.6054 (5)	0.6374 (4)	0.0354 (12)	
H49	0.247931	0.651035	0.648040	0.042*	
C50	0.31255 (15)	0.6426 (4)	0.6521 (3)	0.0252 (9)	
C51	0.3635 (2)	0.3893 (4)	0.5925 (4)	0.0349 (11)	
H51	0.392547	0.426363	0.602465	0.042*	
C52	0.3713 (3)	0.2997 (5)	0.6566 (5)	0.0515 (17)	
H52A	0.375328	0.329392	0.712378	0.077*	
H52B	0.398046	0.260064	0.658719	0.077*	
H52C	0.345406	0.252199	0.639885	0.077*	
C53	0.3473 (2)	0.3454 (5)	0.5018 (4)	0.0441 (14)	
H53A	0.339329	0.404357	0.461655	0.066*	
H53B	0.321086	0.300420	0.493272	0.066*	
H53C	0.371194	0.303714	0.492801	0.066*	
C54	0.32659 (17)	0.7519 (4)	0.6884 (3)	0.0286 (9)	
H54	0.357329	0.765947	0.687678	0.034*	
C55	0.3285 (2)	0.7536 (6)	0.7818 (4)	0.0422 (13)	
H55A	0.349082	0.698622	0.813622	0.063*	
H55B	0.298639	0.740184	0.783963	0.063*	
H55C	0.339020	0.822963	0.806553	0.063*	
C56	0.2958 (2)	0.8386 (5)	0.6375 (5)	0.0445 (14)	
H56A	0.296648	0.839253	0.580044	0.067*	
H56B	0.305648	0.907309	0.664184	0.067*	
H56C	0.265159	0.824765	0.635536	0.067*	

### Atomic displacement parameters (Å^2^)

**Table:**

	*U*^11^	*U*^22^	*U*^33^	*U*^12^	*U*^13^	*U*^23^
Mn1	0.0168 (3)	0.0254 (3)	0.0250 (3)	−0.0019 (2)	0.0087 (2)	−0.0027 (3)
O1	0.0203 (15)	0.046 (2)	0.0307 (18)	−0.0062 (15)	0.0115 (14)	−0.0132 (16)
C1	0.023 (2)	0.075 (5)	0.040 (3)	−0.013 (3)	0.007 (2)	−0.029 (3)
O2	0.0208 (15)	0.044 (2)	0.037 (2)	−0.0079 (14)	0.0156 (14)	−0.0157 (16)
C2	0.028 (2)	0.044 (3)	0.041 (3)	−0.012 (2)	0.013 (2)	−0.017 (2)
O3	0.0301 (19)	0.043 (2)	0.048 (2)	−0.0038 (17)	0.0073 (17)	0.0173 (19)
C3	0.034 (3)	0.070 (5)	0.040 (3)	0.011 (3)	0.005 (2)	0.019 (3)
O4	0.0324 (18)	0.0347 (19)	0.036 (2)	−0.0044 (15)	0.0143 (15)	−0.0090 (15)
C4	0.048 (3)	0.052 (4)	0.038 (3)	−0.018 (3)	0.020 (3)	−0.015 (3)
O5	0.0211 (15)	0.0308 (19)	0.047 (2)	0.0036 (13)	0.0093 (15)	0.0033 (16)
C5	0.025 (2)	0.053 (4)	0.057 (4)	0.012 (2)	0.005 (2)	−0.006 (3)
O6	0.061 (3)	0.047 (3)	0.037 (2)	−0.008 (2)	0.007 (2)	0.0022 (19)
C6	0.067 (5)	0.064 (5)	0.076 (6)	0.001 (4)	0.016 (4)	0.028 (5)
O7	0.073 (4)	0.034 (2)	0.059 (3)	−0.003 (2)	0.024 (3)	0.006 (2)
C7	0.079 (7)	0.049 (5)	0.150 (11)	−0.008 (4)	0.061 (7)	0.009 (6)
O8	0.119 (6)	0.057 (3)	0.039 (3)	−0.001 (3)	0.020 (3)	−0.014 (2)
C8	0.094 (7)	0.052 (4)	0.058 (5)	0.017 (4)	0.013 (5)	−0.019 (4)
P1	0.0157 (4)	0.0217 (5)	0.0178 (4)	−0.0022 (4)	0.0048 (4)	0.0031 (4)
O9	0.0177 (13)	0.0232 (15)	0.0213 (14)	−0.0026 (11)	0.0072 (11)	0.0031 (11)
O10	0.0245 (15)	0.0229 (16)	0.0292 (17)	0.0019 (12)	0.0019 (13)	0.0036 (13)
O11	0.0186 (13)	0.0254 (16)	0.0239 (15)	−0.0050 (11)	0.0061 (11)	0.0035 (12)
O12	0.0161 (13)	0.0243 (15)	0.0227 (14)	−0.0013 (11)	0.0078 (11)	−0.0006 (12)
C9	0.0180 (18)	0.028 (2)	0.029 (2)	−0.0085 (16)	0.0077 (16)	−0.0026 (17)
C10	0.024 (2)	0.027 (2)	0.045 (3)	−0.0078 (18)	0.010 (2)	0.001 (2)
C11	0.042 (3)	0.032 (3)	0.057 (4)	−0.008 (2)	0.016 (3)	−0.012 (3)
C12	0.044 (3)	0.050 (4)	0.037 (3)	−0.014 (3)	0.010 (2)	−0.019 (3)
C13	0.036 (3)	0.042 (3)	0.026 (2)	−0.011 (2)	0.005 (2)	−0.006 (2)
C14	0.0219 (19)	0.029 (2)	0.022 (2)	−0.0104 (17)	0.0038 (16)	−0.0028 (17)
C15	0.048 (3)	0.022 (2)	0.052 (4)	−0.002 (2)	0.011 (3)	0.002 (2)
C16	0.092 (7)	0.066 (6)	0.084 (7)	−0.045 (5)	0.001 (5)	0.027 (5)
C17	0.069 (6)	0.139 (11)	0.085 (7)	0.066 (7)	0.020 (6)	0.029 (7)
C18	0.024 (2)	0.031 (2)	0.022 (2)	−0.0055 (18)	−0.0037 (17)	0.0023 (17)
C19	0.024 (2)	0.043 (3)	0.032 (3)	−0.003 (2)	0.0016 (19)	0.000 (2)
C20	0.035 (3)	0.051 (4)	0.033 (3)	−0.002 (2)	−0.001 (2)	0.012 (2)
C21	0.0209 (19)	0.026 (2)	0.0222 (19)	−0.0022 (16)	0.0078 (16)	0.0014 (16)
C22	0.023 (2)	0.033 (2)	0.027 (2)	−0.0052 (17)	0.0107 (17)	0.0023 (18)
C23	0.022 (2)	0.060 (4)	0.037 (3)	−0.003 (2)	0.016 (2)	−0.001 (3)
C24	0.024 (2)	0.070 (4)	0.037 (3)	0.012 (3)	0.012 (2)	0.001 (3)
C25	0.035 (3)	0.047 (3)	0.035 (3)	0.016 (2)	0.012 (2)	0.004 (2)
C26	0.030 (2)	0.029 (2)	0.026 (2)	0.0059 (18)	0.0110 (18)	0.0014 (17)
C27	0.030 (2)	0.038 (3)	0.033 (2)	−0.010 (2)	0.016 (2)	0.003 (2)
C28	0.062 (4)	0.041 (4)	0.055 (4)	−0.021 (3)	0.012 (3)	−0.002 (3)
C29	0.058 (4)	0.057 (4)	0.033 (3)	−0.011 (3)	0.022 (3)	0.011 (3)
C30	0.043 (3)	0.022 (2)	0.035 (3)	0.004 (2)	0.021 (2)	0.0016 (18)
C31	0.072 (5)	0.040 (3)	0.037 (3)	−0.006 (3)	0.019 (3)	0.007 (3)
C32	0.046 (3)	0.046 (3)	0.036 (3)	−0.014 (3)	0.012 (3)	−0.004 (2)
P2	0.0128 (4)	0.0282 (5)	0.0168 (4)	0.0042 (4)	0.0039 (3)	−0.0041 (4)
O13	0.0234 (15)	0.0327 (18)	0.0260 (16)	0.0015 (13)	0.0094 (13)	−0.0055 (13)
O14	0.0175 (14)	0.0394 (19)	0.0254 (16)	−0.0052 (13)	0.0064 (12)	0.0007 (14)
O15	0.0237 (15)	0.0281 (17)	0.0286 (17)	0.0090 (13)	0.0090 (13)	−0.0023 (13)
O16	0.0135 (12)	0.0206 (14)	0.0271 (15)	0.0021 (11)	0.0058 (11)	−0.0010 (12)
C33	0.024 (2)	0.018 (2)	0.034 (2)	0.0052 (16)	0.0073 (18)	0.0037 (17)
C34	0.029 (2)	0.017 (2)	0.061 (4)	0.0064 (18)	0.009 (2)	0.001 (2)
C35	0.045 (3)	0.025 (3)	0.079 (5)	0.008 (2)	0.019 (3)	0.019 (3)
C36	0.069 (5)	0.043 (4)	0.061 (5)	0.019 (3)	0.019 (4)	0.029 (3)
C37	0.059 (4)	0.042 (3)	0.035 (3)	0.011 (3)	−0.008 (3)	0.012 (3)
C38	0.030 (2)	0.028 (2)	0.033 (3)	0.0063 (19)	−0.001 (2)	0.007 (2)
C39	0.053 (4)	0.022 (3)	0.085 (5)	−0.006 (3)	0.005 (4)	−0.017 (3)
C40A	0.090 (11)	0.083 (13)	0.086 (13)	−0.035 (10)	0.046 (10)	−0.033 (10)
C41A	0.045 (9)	0.074 (11)	0.114 (14)	−0.013 (8)	0.016 (9)	−0.040 (10)
C40B	0.077 (12)	0.058 (13)	0.109 (17)	−0.018 (10)	0.035 (13)	−0.049 (12)
C41B	0.032 (9)	0.080 (14)	0.126 (18)	0.016 (9)	0.004 (10)	−0.032 (13)
C42	0.026 (2)	0.027 (2)	0.038 (3)	−0.0010 (18)	−0.010 (2)	0.007 (2)
C43	0.030 (3)	0.043 (3)	0.069 (5)	0.002 (2)	0.002 (3)	−0.021 (3)
C44	0.040 (3)	0.040 (3)	0.035 (3)	−0.005 (2)	0.001 (2)	−0.002 (2)
C45	0.0149 (17)	0.022 (2)	0.032 (2)	−0.0008 (15)	0.0068 (16)	−0.0058 (17)
C46	0.024 (2)	0.021 (2)	0.039 (3)	−0.0026 (16)	0.0108 (19)	−0.0031 (18)
C47	0.028 (2)	0.035 (3)	0.044 (3)	−0.012 (2)	0.009 (2)	−0.003 (2)
C48	0.018 (2)	0.047 (3)	0.052 (3)	−0.009 (2)	0.012 (2)	0.004 (3)
C49	0.019 (2)	0.041 (3)	0.050 (3)	0.0006 (19)	0.018 (2)	−0.003 (2)
C50	0.0165 (18)	0.027 (2)	0.033 (2)	0.0000 (16)	0.0099 (16)	−0.0028 (18)
C51	0.039 (3)	0.022 (2)	0.041 (3)	0.005 (2)	0.010 (2)	−0.003 (2)
C52	0.067 (5)	0.033 (3)	0.057 (4)	0.016 (3)	0.025 (3)	0.013 (3)
C53	0.053 (4)	0.031 (3)	0.047 (3)	0.003 (3)	0.014 (3)	−0.003 (2)
C54	0.030 (2)	0.025 (2)	0.032 (2)	0.0048 (18)	0.0130 (19)	−0.0057 (18)
C55	0.047 (3)	0.045 (3)	0.040 (3)	0.004 (3)	0.023 (3)	−0.008 (3)
C56	0.048 (3)	0.027 (3)	0.053 (4)	0.011 (2)	0.010 (3)	−0.005 (2)

### Geometric parameters (Å, º)

**Table:**

Mn1—O1	2.146 (3)	C27—C28	1.522 (9)
Mn1—O2	2.236 (4)	C27—C29	1.527 (8)
Mn1—O3	2.158 (4)	C27—H27	1.0000
Mn1—O4	2.213 (4)	C28—H28A	0.9800
Mn1—O5	2.220 (4)	C28—H28B	0.9800
Mn1—O9	2.116 (3)	C28—H28C	0.9800
O1—C1	1.430 (6)	C29—H29A	0.9800
O1—H1	0.85 (3)	C29—H29B	0.9800
C1—H1A	0.9800	C29—H29C	0.9800
C1—H1B	0.9800	C30—C32	1.522 (8)
C1—H1C	0.9800	C30—C31	1.523 (8)
O2—C2	1.443 (6)	C30—H30	1.0000
O2—H2	0.82 (3)	C31—H31A	0.9800
C2—H2A	0.9800	C31—H31B	0.9800
C2—H2B	0.9800	C31—H31C	0.9800
C2—H2C	0.9800	C32—H32A	0.9800
O3—C3	1.432 (8)	C32—H32B	0.9800
O3—H3	0.86 (3)	C32—H32C	0.9800
C3—H3A	0.9800	P2—O13	1.496 (4)
C3—H3B	0.9800	P2—O14	1.488 (3)
C3—H3C	0.9800	P2—O15	1.607 (3)
O4—C4	1.448 (8)	P2—O16	1.600 (3)
O4—H4	0.85 (3)	O15—C33	1.397 (6)
C4—H4A	0.9800	O16—C45	1.409 (5)
C4—H4B	0.9800	C33—C38	1.394 (7)
C4—H4C	0.9800	C33—C34	1.405 (7)
O5—C5	1.416 (7)	C34—C35	1.397 (10)
O5—H5	0.85 (3)	C34—C39	1.506 (10)
C5—H5A	0.9800	C35—C36	1.360 (12)
C5—H5B	0.9800	C35—H35	0.9500
C5—H5C	0.9800	C36—C37	1.392 (11)
O6—C6	1.408 (10)	C36—H36	0.9500
O6—H6	0.86 (3)	C37—C38	1.405 (8)
C6—H6A	0.9800	C37—H37	0.9500
C6—H6B	0.9800	C38—C42	1.513 (8)
C6—H6C	0.9800	C39—C41B	1.473 (13)
O7—C7	1.419 (11)	C39—C40A	1.482 (13)
O7—H7	0.85 (3)	C39—C40B	1.504 (13)
C7—H7A	0.9800	C39—C41A	1.505 (13)
C7—H7B	0.9800	C39—H39A	1.0000
C7—H7C	0.9800	C39—H39B	1.0000
O8—C8	1.377 (11)	C40A—H40A	0.9800
O8—H8	0.85 (3)	C40A—H40B	0.9800
C8—H8A	0.9800	C40A—H40C	0.9800
C8—H8B	0.9800	C41A—H41A	0.9800
C8—H8C	0.9800	C41A—H41B	0.9800
P1—O9	1.503 (3)	C41A—H41C	0.9800
P1—O10	1.488 (3)	C40B—H40D	0.9800
P1—O11	1.600 (3)	C40B—H40E	0.9800
P1—O12	1.597 (3)	C40B—H40F	0.9800
O11—C9	1.410 (6)	C41B—H41D	0.9800
O12—C21	1.403 (5)	C41B—H41E	0.9800
C9—C10	1.392 (8)	C41B—H41F	0.9800
C9—C14	1.415 (7)	C42—C44	1.515 (9)
C10—C11	1.404 (9)	C42—C43	1.547 (9)
C10—C15	1.517 (9)	C42—H42	1.0000
C11—C12	1.391 (10)	C43—H43A	0.9800
C11—H11	0.9500	C43—H43B	0.9800
C12—C13	1.392 (10)	C43—H43C	0.9800
C12—H12	0.9500	C44—H44A	0.9800
C13—C14	1.387 (7)	C44—H44B	0.9800
C13—H13	0.9500	C44—H44C	0.9800
C14—C18	1.493 (8)	C45—C46	1.408 (6)
C15—C17	1.494 (12)	C45—C50	1.414 (6)
C15—C16	1.523 (10)	C46—C47	1.401 (7)
C15—H15	1.0000	C46—C51	1.511 (7)
C16—H16A	0.9800	C47—C48	1.389 (9)
C16—H16B	0.9800	C47—H47	0.9500
C16—H16C	0.9800	C48—C49	1.380 (9)
C17—H17A	0.9800	C48—H48	0.9500
C17—H17B	0.9800	C49—C50	1.402 (6)
C17—H17C	0.9800	C49—H49	0.9500
C18—C19	1.528 (8)	C50—C54	1.517 (7)
C18—C20	1.529 (8)	C51—C52	1.527 (9)
C18—H18	1.0000	C51—C53	1.542 (9)
C19—H19A	0.9800	C51—H51	1.0000
C19—H19B	0.9800	C52—H52A	0.9800
C19—H19C	0.9800	C52—H52B	0.9800
C20—H20A	0.9800	C52—H52C	0.9800
C20—H20B	0.9800	C53—H53A	0.9800
C20—H20C	0.9800	C53—H53B	0.9800
C21—C22	1.387 (6)	C53—H53C	0.9800
C21—C26	1.389 (7)	C54—C56	1.525 (8)
C22—C23	1.397 (7)	C54—C55	1.556 (8)
C22—C27	1.512 (8)	C54—H54	1.0000
C23—C24	1.370 (10)	C55—H55A	0.9800
C23—H23	0.9500	C55—H55B	0.9800
C24—C25	1.385 (10)	C55—H55C	0.9800
C24—H24	0.9500	C56—H56A	0.9800
C25—C26	1.390 (7)	C56—H56B	0.9800
C25—H25	0.9500	C56—H56C	0.9800
C26—C30	1.519 (8)		
			
O9—Mn1—O1	176.85 (14)	C28—C27—H27	108.4
O9—Mn1—O3	89.81 (14)	C29—C27—H27	108.4
O1—Mn1—O3	92.26 (16)	C27—C28—H28A	109.5
O9—Mn1—O4	90.71 (14)	C27—C28—H28B	109.5
O1—Mn1—O4	86.88 (14)	H28A—C28—H28B	109.5
O3—Mn1—O4	91.29 (18)	C27—C28—H28C	109.5
O9—Mn1—O5	88.84 (13)	H28A—C28—H28C	109.5
O1—Mn1—O5	89.09 (15)	H28B—C28—H28C	109.5
O3—Mn1—O5	178.62 (16)	C27—C29—H29A	109.5
O4—Mn1—O5	89.03 (16)	C27—C29—H29B	109.5
O9—Mn1—O2	93.86 (13)	H29A—C29—H29B	109.5
O1—Mn1—O2	88.51 (13)	C27—C29—H29C	109.5
O3—Mn1—O2	89.98 (18)	H29A—C29—H29C	109.5
O4—Mn1—O2	175.26 (14)	H29B—C29—H29C	109.5
O5—Mn1—O2	89.81 (16)	C26—C30—C32	110.4 (4)
C1—O1—Mn1	125.4 (3)	C26—C30—C31	112.6 (5)
C1—O1—H1	109.5	C32—C30—C31	110.8 (5)
Mn1—O1—H1	103.8	C26—C30—H30	107.6
O1—C1—H1A	109.5	C32—C30—H30	107.6
O1—C1—H1B	109.5	C31—C30—H30	107.6
H1A—C1—H1B	109.5	C30—C31—H31A	109.5
O1—C1—H1C	109.5	C30—C31—H31B	109.5
H1A—C1—H1C	109.5	H31A—C31—H31B	109.5
H1B—C1—H1C	109.5	C30—C31—H31C	109.5
C2—O2—Mn1	123.8 (3)	H31A—C31—H31C	109.5
C2—O2—H2	109.5	H31B—C31—H31C	109.5
Mn1—O2—H2	117.6	C30—C32—H32A	109.5
O2—C2—H2A	109.5	C30—C32—H32B	109.5
O2—C2—H2B	109.5	H32A—C32—H32B	109.5
H2A—C2—H2B	109.5	C30—C32—H32C	109.5
O2—C2—H2C	109.5	H32A—C32—H32C	109.5
H2A—C2—H2C	109.5	H32B—C32—H32C	109.5
H2B—C2—H2C	109.5	O14—P2—O13	117.1 (2)
C3—O3—Mn1	120.8 (4)	O14—P2—O16	105.98 (18)
C3—O3—H3	109.5	O13—P2—O16	111.4 (2)
Mn1—O3—H3	122.2	O14—P2—O15	112.0 (2)
O3—C3—H3A	109.5	O13—P2—O15	109.5 (2)
O3—C3—H3B	109.5	O16—P2—O15	99.33 (18)
H3A—C3—H3B	109.5	C33—O15—P2	123.4 (3)
O3—C3—H3C	109.5	C45—O16—P2	122.9 (3)
H3A—C3—H3C	109.5	C38—C33—O15	117.9 (4)
H3B—C3—H3C	109.5	C38—C33—C34	123.6 (5)
C4—O4—Mn1	125.3 (4)	O15—C33—C34	118.4 (5)
C4—O4—H4	109.5	C35—C34—C33	117.4 (6)
Mn1—O4—H4	125.0	C35—C34—C39	120.2 (6)
O4—C4—H4A	109.5	C33—C34—C39	122.4 (6)
O4—C4—H4B	109.5	C36—C35—C34	120.7 (6)
H4A—C4—H4B	109.5	C36—C35—H35	119.6
O4—C4—H4C	109.5	C34—C35—H35	119.6
H4A—C4—H4C	109.5	C35—C36—C37	121.0 (7)
H4B—C4—H4C	109.5	C35—C36—H36	119.5
C5—O5—Mn1	125.9 (4)	C37—C36—H36	119.5
C5—O5—H5	109.5	C36—C37—C38	121.1 (7)
Mn1—O5—H5	102.9	C36—C37—H37	119.4
O5—C5—H5A	109.5	C38—C37—H37	119.4
O5—C5—H5B	109.5	C33—C38—C37	116.1 (5)
H5A—C5—H5B	109.5	C33—C38—C42	122.8 (5)
O5—C5—H5C	109.5	C37—C38—C42	121.0 (5)
H5A—C5—H5C	109.5	C41B—C39—C40B	112.9 (17)
H5B—C5—H5C	109.5	C40A—C39—C41A	112.9 (13)
C6—O6—H6	109.5	C41B—C39—C34	123.2 (16)
O6—C6—H6A	109.5	C40A—C39—C34	117.1 (13)
O6—C6—H6B	109.5	C40B—C39—C34	106.7 (15)
H6A—C6—H6B	109.5	C41A—C39—C34	109.9 (11)
O6—C6—H6C	109.5	C40A—C39—H39A	105.3
H6A—C6—H6C	109.5	C41A—C39—H39A	105.3
H6B—C6—H6C	109.5	C34—C39—H39A	105.3
C7—O7—H7	109.5	C41B—C39—H39B	104.0
O7—C7—H7A	109.5	C40B—C39—H39B	104.0
O7—C7—H7B	109.5	C34—C39—H39B	104.0
H7A—C7—H7B	109.5	C39—C40A—H40A	109.5
O7—C7—H7C	109.5	C39—C40A—H40B	109.5
H7A—C7—H7C	109.5	H40A—C40A—H40B	109.5
H7B—C7—H7C	109.5	C39—C40A—H40C	109.5
C8—O8—H8	109.5	H40A—C40A—H40C	109.5
O8—C8—H8A	109.5	H40B—C40A—H40C	109.5
O8—C8—H8B	109.5	C39—C41A—H41A	109.5
H8A—C8—H8B	109.5	C39—C41A—H41B	109.5
O8—C8—H8C	109.5	H41A—C41A—H41B	109.5
H8A—C8—H8C	109.5	C39—C41A—H41C	109.5
H8B—C8—H8C	109.5	H41A—C41A—H41C	109.5
O10—P1—O9	115.2 (2)	H41B—C41A—H41C	109.5
O10—P1—O12	105.8 (2)	C39—C40B—H40D	109.5
O9—P1—O12	112.90 (18)	C39—C40B—H40E	109.5
O10—P1—O11	112.08 (19)	H40D—C40B—H40E	109.5
O9—P1—O11	109.62 (18)	C39—C40B—H40F	109.5
O12—P1—O11	100.29 (18)	H40D—C40B—H40F	109.5
P1—O9—Mn1	132.20 (19)	H40E—C40B—H40F	109.5
C9—O11—P1	122.4 (3)	C39—C41B—H41D	109.5
C21—O12—P1	127.0 (3)	C39—C41B—H41E	109.5
C10—C9—O11	119.1 (5)	H41D—C41B—H41E	109.5
C10—C9—C14	123.5 (5)	C39—C41B—H41F	109.5
O11—C9—C14	117.3 (4)	H41D—C41B—H41F	109.5
C9—C10—C11	116.8 (5)	H41E—C41B—H41F	109.5
C9—C10—C15	122.4 (5)	C38—C42—C44	109.9 (5)
C11—C10—C15	120.7 (5)	C38—C42—C43	112.3 (6)
C12—C11—C10	121.2 (6)	C44—C42—C43	110.5 (5)
C12—C11—H11	119.4	C38—C42—H42	108.0
C10—C11—H11	119.4	C44—C42—H42	108.0
C11—C12—C13	120.1 (6)	C43—C42—H42	108.0
C11—C12—H12	119.9	C42—C43—H43A	109.5
C13—C12—H12	119.9	C42—C43—H43B	109.5
C14—C13—C12	121.1 (6)	H43A—C43—H43B	109.5
C14—C13—H13	119.4	C42—C43—H43C	109.5
C12—C13—H13	119.4	H43A—C43—H43C	109.5
C13—C14—C9	117.1 (5)	H43B—C43—H43C	109.5
C13—C14—C18	121.5 (5)	C42—C44—H44A	109.5
C9—C14—C18	121.3 (4)	C42—C44—H44B	109.5
C17—C15—C10	112.8 (7)	H44A—C44—H44B	109.5
C17—C15—C16	113.0 (9)	C42—C44—H44C	109.5
C10—C15—C16	110.9 (6)	H44A—C44—H44C	109.5
C17—C15—H15	106.6	H44B—C44—H44C	109.5
C10—C15—H15	106.6	C46—C45—O16	119.3 (4)
C16—C15—H15	106.6	C46—C45—C50	122.8 (4)
C15—C16—H16A	109.5	O16—C45—C50	117.8 (4)
C15—C16—H16B	109.5	C47—C46—C45	117.0 (5)
H16A—C16—H16B	109.5	C47—C46—C51	119.6 (5)
C15—C16—H16C	109.5	C45—C46—C51	123.3 (4)
H16A—C16—H16C	109.5	C48—C47—C46	121.6 (5)
H16B—C16—H16C	109.5	C48—C47—H47	119.2
C15—C17—H17A	109.5	C46—C47—H47	119.2
C15—C17—H17B	109.5	C49—C48—C47	119.8 (5)
H17A—C17—H17B	109.5	C49—C48—H48	120.1
C15—C17—H17C	109.5	C47—C48—H48	120.1
H17A—C17—H17C	109.5	C48—C49—C50	121.9 (5)
H17B—C17—H17C	109.5	C48—C49—H49	119.1
C14—C18—C19	111.0 (4)	C50—C49—H49	119.1
C14—C18—C20	113.6 (5)	C49—C50—C45	116.8 (5)
C19—C18—C20	109.8 (5)	C49—C50—C54	120.9 (4)
C14—C18—H18	107.4	C45—C50—C54	122.2 (4)
C19—C18—H18	107.4	C46—C51—C52	109.9 (5)
C20—C18—H18	107.4	C46—C51—C53	111.5 (5)
C18—C19—H19A	109.5	C52—C51—C53	110.8 (5)
C18—C19—H19B	109.5	C46—C51—H51	108.2
H19A—C19—H19B	109.5	C52—C51—H51	108.2
C18—C19—H19C	109.5	C53—C51—H51	108.2
H19A—C19—H19C	109.5	C51—C52—H52A	109.5
H19B—C19—H19C	109.5	C51—C52—H52B	109.5
C18—C20—H20A	109.5	H52A—C52—H52B	109.5
C18—C20—H20B	109.5	C51—C52—H52C	109.5
H20A—C20—H20B	109.5	H52A—C52—H52C	109.5
C18—C20—H20C	109.5	H52B—C52—H52C	109.5
H20A—C20—H20C	109.5	C51—C53—H53A	109.5
H20B—C20—H20C	109.5	C51—C53—H53B	109.5
C22—C21—C26	123.2 (5)	H53A—C53—H53B	109.5
C22—C21—O12	118.3 (4)	C51—C53—H53C	109.5
C26—C21—O12	118.3 (4)	H53A—C53—H53C	109.5
C21—C22—C23	116.6 (5)	H53B—C53—H53C	109.5
C21—C22—C27	122.6 (4)	C50—C54—C56	112.5 (5)
C23—C22—C27	120.8 (5)	C50—C54—C55	109.0 (5)
C24—C23—C22	122.4 (5)	C56—C54—C55	110.9 (5)
C24—C23—H23	118.8	C50—C54—H54	108.1
C22—C23—H23	118.8	C56—C54—H54	108.1
C23—C24—C25	119.0 (5)	C55—C54—H54	108.1
C23—C24—H24	120.5	C54—C55—H55A	109.5
C25—C24—H24	120.5	C54—C55—H55B	109.5
C24—C25—C26	121.4 (6)	H55A—C55—H55B	109.5
C24—C25—H25	119.3	C54—C55—H55C	109.5
C26—C25—H25	119.3	H55A—C55—H55C	109.5
C21—C26—C25	117.4 (5)	H55B—C55—H55C	109.5
C21—C26—C30	121.9 (4)	C54—C56—H56A	109.5
C25—C26—C30	120.5 (5)	C54—C56—H56B	109.5
C22—C27—C28	111.2 (5)	H56A—C56—H56B	109.5
C22—C27—C29	110.1 (5)	C54—C56—H56C	109.5
C28—C27—C29	110.4 (5)	H56A—C56—H56C	109.5
C22—C27—H27	108.4	H56B—C56—H56C	109.5
			
O10—P1—O9—Mn1	25.7 (3)	O13—P2—O15—C33	−45.8 (4)
O12—P1—O9—Mn1	−95.9 (3)	O16—P2—O15—C33	−162.6 (4)
O11—P1—O9—Mn1	153.2 (2)	O14—P2—O16—C45	166.3 (3)
O10—P1—O11—C9	90.7 (4)	O13—P2—O16—C45	−65.3 (4)
O9—P1—O11—C9	−38.5 (4)	O15—P2—O16—C45	50.1 (4)
O12—P1—O11—C9	−157.5 (3)	P2—O15—C33—C38	88.9 (5)
O10—P1—O12—C21	146.7 (4)	P2—O15—C33—C34	−94.9 (5)
O9—P1—O12—C21	−86.5 (4)	C38—C33—C34—C35	−1.8 (8)
O11—P1—O12—C21	30.1 (4)	O15—C33—C34—C35	−177.8 (5)
P1—O11—C9—C10	−88.0 (5)	C38—C33—C34—C39	175.1 (5)
P1—O11—C9—C14	95.9 (4)	O15—C33—C34—C39	−1.0 (8)
O11—C9—C10—C11	−177.9 (4)	C33—C34—C35—C36	−0.4 (10)
C14—C9—C10—C11	−2.2 (7)	C39—C34—C35—C36	−177.3 (7)
O11—C9—C10—C15	−1.1 (7)	C34—C35—C36—C37	1.7 (12)
C14—C9—C10—C15	174.7 (5)	C35—C36—C37—C38	−1.0 (12)
C9—C10—C11—C12	−0.4 (8)	O15—C33—C38—C37	178.5 (5)
C15—C10—C11—C12	−177.3 (6)	C34—C33—C38—C37	2.4 (8)
C10—C11—C12—C13	1.6 (10)	O15—C33—C38—C42	1.3 (8)
C11—C12—C13—C14	−0.3 (9)	C34—C33—C38—C42	−174.8 (5)
C12—C13—C14—C9	−2.1 (8)	C36—C37—C38—C33	−1.0 (10)
C12—C13—C14—C18	175.2 (5)	C36—C37—C38—C42	176.2 (7)
C10—C9—C14—C13	3.4 (7)	C35—C34—C39—C41B	−61 (2)
O11—C9—C14—C13	179.2 (4)	C33—C34—C39—C41B	122 (2)
C10—C9—C14—C18	−173.9 (4)	C35—C34—C39—C40A	98.1 (18)
O11—C9—C14—C18	1.9 (6)	C33—C34—C39—C40A	−78.7 (17)
C9—C10—C15—C17	125.9 (8)	C35—C34—C39—C40B	72 (2)
C11—C10—C15—C17	−57.4 (10)	C33—C34—C39—C40B	−105 (2)
C9—C10—C15—C16	−106.3 (8)	C35—C34—C39—C41A	−32.5 (15)
C11—C10—C15—C16	70.4 (9)	C33—C34—C39—C41A	150.7 (14)
C13—C14—C18—C19	−102.7 (5)	C33—C38—C42—C44	85.4 (6)
C9—C14—C18—C19	74.5 (5)	C37—C38—C42—C44	−91.7 (7)
C13—C14—C18—C20	21.6 (7)	C33—C38—C42—C43	−151.2 (5)
C9—C14—C18—C20	−161.2 (4)	C37—C38—C42—C43	31.8 (8)
P1—O12—C21—C22	−90.2 (5)	P2—O16—C45—C46	86.4 (5)
P1—O12—C21—C26	94.9 (5)	P2—O16—C45—C50	−96.5 (5)
C26—C21—C22—C23	−2.2 (8)	O16—C45—C46—C47	179.4 (5)
O12—C21—C22—C23	−176.9 (4)	C50—C45—C46—C47	2.5 (8)
C26—C21—C22—C27	177.6 (5)	O16—C45—C46—C51	1.9 (8)
O12—C21—C22—C27	2.9 (7)	C50—C45—C46—C51	−175.0 (5)
C21—C22—C23—C24	1.1 (8)	C45—C46—C47—C48	−2.0 (9)
C27—C22—C23—C24	−178.7 (6)	C51—C46—C47—C48	175.6 (6)
C22—C23—C24—C25	0.1 (9)	C46—C47—C48—C49	1.0 (10)
C23—C24—C25—C26	−0.4 (9)	C47—C48—C49—C50	−0.4 (10)
C22—C21—C26—C25	2.0 (8)	C48—C49—C50—C45	0.8 (9)
O12—C21—C26—C25	176.6 (5)	C48—C49—C50—C54	−177.1 (6)
C22—C21—C26—C30	−174.6 (5)	C46—C45—C50—C49	−1.9 (8)
O12—C21—C26—C30	0.1 (7)	O16—C45—C50—C49	−178.9 (5)
C24—C25—C26—C21	−0.6 (8)	C46—C45—C50—C54	176.0 (5)
C24—C25—C26—C30	176.0 (5)	O16—C45—C50—C54	−1.0 (7)
C21—C22—C27—C28	124.6 (6)	C47—C46—C51—C52	−64.9 (7)
C23—C22—C27—C28	−55.6 (7)	C45—C46—C51—C52	112.5 (6)
C21—C22—C27—C29	−112.7 (6)	C47—C46—C51—C53	58.4 (7)
C23—C22—C27—C29	67.1 (7)	C45—C46—C51—C53	−124.2 (6)
C21—C26—C30—C32	91.5 (6)	C49—C50—C54—C56	−54.2 (7)
C25—C26—C30—C32	−85.0 (7)	C45—C50—C54—C56	127.9 (6)
C21—C26—C30—C31	−144.2 (5)	C49—C50—C54—C55	69.1 (6)
C25—C26—C30—C31	39.4 (7)	C45—C50—C54—C55	−108.7 (6)
O14—P2—O15—C33	85.9 (4)		

### Hydrogen-bond geometry (Å, º)

**Table:**

*D*—H···*A*	*D*—H	H···*A*	*D*···*A*	*D*—H···*A*
O1—H1···O13	0.85	1.79	2.537 (5)	145
O2—H2···O14	0.82	1.99	2.724 (5)	148
O3—H3···O7	0.86	1.79	2.644 (6)	170
O4—H4···O6	0.85	1.95	2.700 (7)	147
O5—H5···O10	0.85	1.84	2.661 (5)	163
O6—H6···O14^i^	0.86	1.89	2.708 (6)	157
O7—H7···O8	0.85	1.88	2.697 (8)	159
O8—H8···O10^ii^	0.85	1.86	2.708 (7)	174
C1—H1*B*···O4	0.98	2.49	3.162 (7)	125
C7—H7*B*···O1	0.98	2.66	3.570 (13)	154

Symmetry codes: (i) *x*, −*y*+1, *z*−1/2; (ii) *x*, −*y*+1, *z*+1/2.
