# Supplementary material for: [Bis(2,6-diiso­propyl­phen­yl) phosphato-κO]pentakis­(methanol-κO)manganese bis­(2,6-diiso­propyl­phen­yl) phosphate methanol tris­olvate
Source: Acta Crystallogr E Crystallogr Commun. 2018 Sep 14;74(Pt 10):1433–8. doi: 10.1107/S2056989018012859 (PMC6176441; doi:10.1107/S2056989018012859)
Supplement: Supplementary file 4 [file e-74-01433-sup5.pdf]

Supporting information

Single crystal X-ray diffraction studies

Since the studied crystal had problems, four additional attempts to prepare crystal batches of $\{[Mn\{OOP(O\text{-}2,6\text{-}^{i}Pr_2C_6H_3)_2\}(CH_3OH)_5]^{+}[OOP(O\text{-}2,6\text{-}^{i}Pr_2C_6H_3)_2]^{-}(CH_3OH)_3\}_{\infty}$ were made, starting either from $Mn(NO_3)_2(H_2O)_6$ or from $MnCl_2(H_2O)_4$ by varying crystal growth conditions. Four attempts to reestablish the crystal structure were done by using different diffractometers [Bruker SMART Platform CCD, 2 different Bruker SMART APEX II instruments) and software (e.g., SHELXTL, SHELXL97, SHELXS-2013, TWINABS, SADABS, CELLNOW, SHELXL-2017). All studied crystals exhibited the *Cc* space group. Integration in monoclinic as well as in triclinic crystal systems were tried. Crystallographic details for two last crystals are presented in Table S1. The problems with crystal structures remain the same, and may be likely caused by some subtle unrevealed twinning.

Table S1. Crystallographic details for two additional crystals of $\{[Mn\{OOP(O\text{-}2,6\text{-}^{i}Pr_2C_6H_3)_2\}(CH_3OH)_5]^{+}[OOP(O\text{-}2,6\text{-}^{i}Pr_2C_6H_3)_2]^{-}(CH_3OH)_3\}_{\infty}$.

	Sample 1	Sample 2
Experimental details		
Chemical formula	$C_{56}H_{100}MnO_{16}P_2$	$C_{56}H_{100}MnO_{16}P_2$
M_r	1146.23	1146.23
Crystal system, space group	Monoclinic, *Cc*	Monoclinic, *Cc*
Temperature (K)	150	150
a, b, c (Å)	31.827 (4), 12.6141 (14), 16.8546 (18)	31.805 (5), 12.5981 (18), 16.840 (2)
β (°)	110.079 (4)	110.074 (3)
V (Å^3)	6355.3 (13)	6337.5 (16)
Z	4	4
Radiation type	Mo Kα	Mo Kα
μ (mm^{-1})	0.319	0.320
Data collection		
Diffractometer	Bruker SMART APEX II	Bruker SMART APEX II
Absorption correction	Multi-scan SADABS (Bruker, 2008)	Multi-scan SADABS (Bruker, 2008)
T_{min}, T_{max}	0.677, 0.839	0.615, 0.747
No. of measured, independent and observed [I > 2σ(I)] reflections	35885, 16355, 12110	64467, 18410, 16235

R_{int}	0.071	0.053
$(\sin \theta/\lambda)_{max}$ (Å^{-1})	0.682	0.703
Refinement		
$R[F^2 > 2\sigma(F^2)]$, $wR(F^2)$, S	0.135, 0.364, 1.32	0.089, 0.241, 1.10
No. of reflections	16355	18410
No. of parameters	730	730
No. of restraints	52	52
H-atom treatment	H-atom parameters constrained	H-atom parameters constrained
$(\Delta/\sigma)_{max}$	0.507	1.001
Δρmax, Δρmin (e Å^{-3})	6.74, −1.22	4.56, −0.91
Absolute structure	Refined as an inversion twin	Refined as an inversion twin
Absolute structure parameter	0.55 (5)	0.47 (3)

Computer programs: APEX2 (Bruker, 2008), SAINT (Bruker, 2008), SHELXS (Sheldrick, 2008), SHELXL2017 (Sheldrick, 2015).

Powder X-ray diffraction studies

Powder patterns were recorded on a Bruker D8 Advance Vario diffractometer, using CuKα_1 radiation. The Rietveld analysis was carried out with the Bruker Topas V5 software (Bruker, 2015).

Crystals of $\{[Mn\{OOP(O\text{-}2,6\text{-}^iPr_2C_6H_3)_2\}(CH_3OH)_5]^+[OOP(O\text{-}2,6\text{-}^iPr_2C_6H_3)_2]^-(CH_3OH)_3\}_\infty$ for powder X-ray diffraction studies remained without a solvent for 6 days in a closed vial. Then the crystals were crashed into smaller pieces for the analysis, which was carried out for four days at room temperature on open air. The sample did not contain a single phase (Fig. S2). Moreover, its phase content was changing during measurements (Fig. S4, S5). After one day, a single phase was detected (Fig. S3), possessing the following unit cell parameters: a = 14.4324(9)Å, b = 14.6838(11)Å, c = 15.6863(10)Å, α = 83.572(2)°, β= 99.078(3)°, γ = 120.365(3)°, V= 2830.3(3) Å^3. Systematic absences are consistent with the space group $P\bar{1}$.

At the beginning of the measurement (Fig. S2), the sample contained the monoclinic phase determined from a single crystal X-ray diffraction experiments (Fig. S1), the found triclinic phase, and at least one unknown phase, which neither was observed in a pure form nor was indexed by using residual peaks. Further but much slower phase transformations took place in the next three days (Fig. S5). However, the stabilization of the phase composition was not yet achieved during measurements.

The all described phase changes can be attributed to facile loss of non-coordinating

methanol molecules and perhaps to some partial hydrolysis on open air.

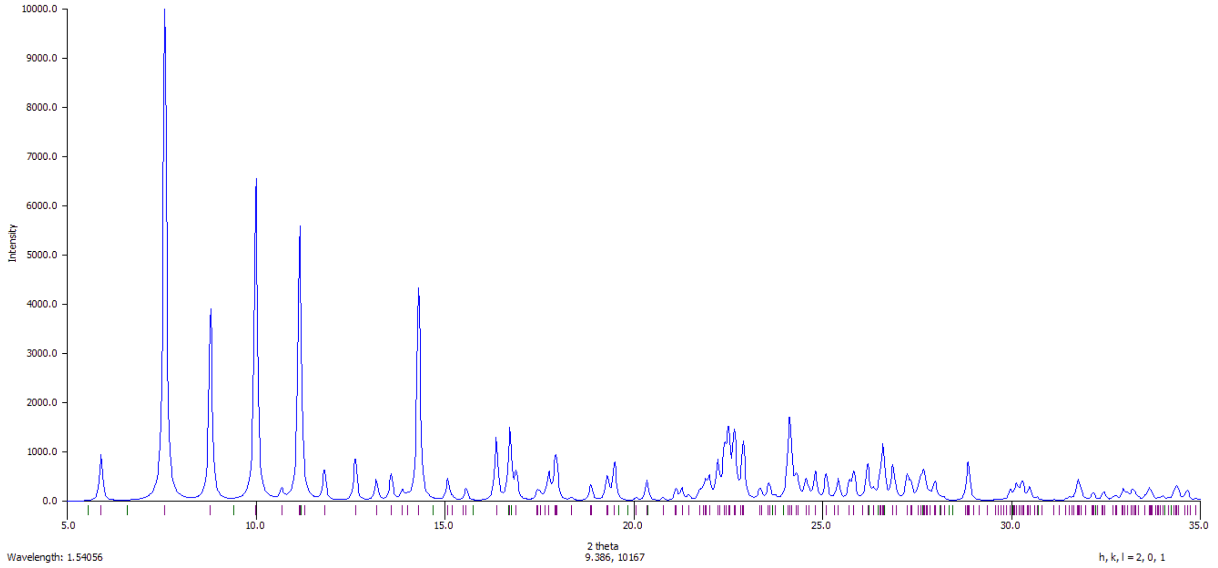

Figure S1. Simulated powder pattern for $\{[Mn\{OOP(O\text{-}2,6\text{-}^{i}Pr_2C_6H_3)_2\}(CH_3OH)_5]^{+}[OOP(O\text{-}2,6\text{-}^{i}Pr_2C_6H_3)_2]^{-}(CH_3OH)_3\}_{\infty}$ with the determined crystal structure (λ=1.54056Å, 2θ=5-35°, Mercury 3.10.1).

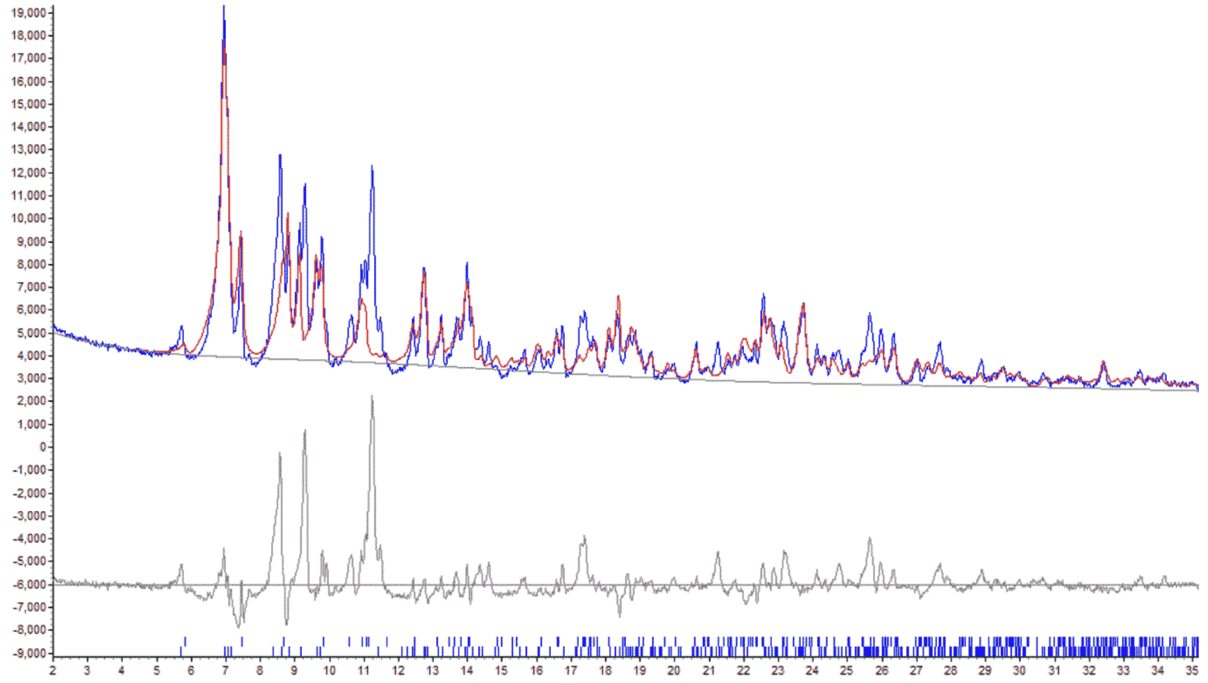

Figure S2. Powder pattern (2θ=2-35°) of the solid in the beginning of the measurements. The experimental (blue), calculated (red) diffraction patterns and the difference curve (gray) clearly demonstrate that modeling by using the assumption of a two-phase system (established monoclinic Cc and proposed triclinic $P\bar{1}$ phases) is unsatisfactory.

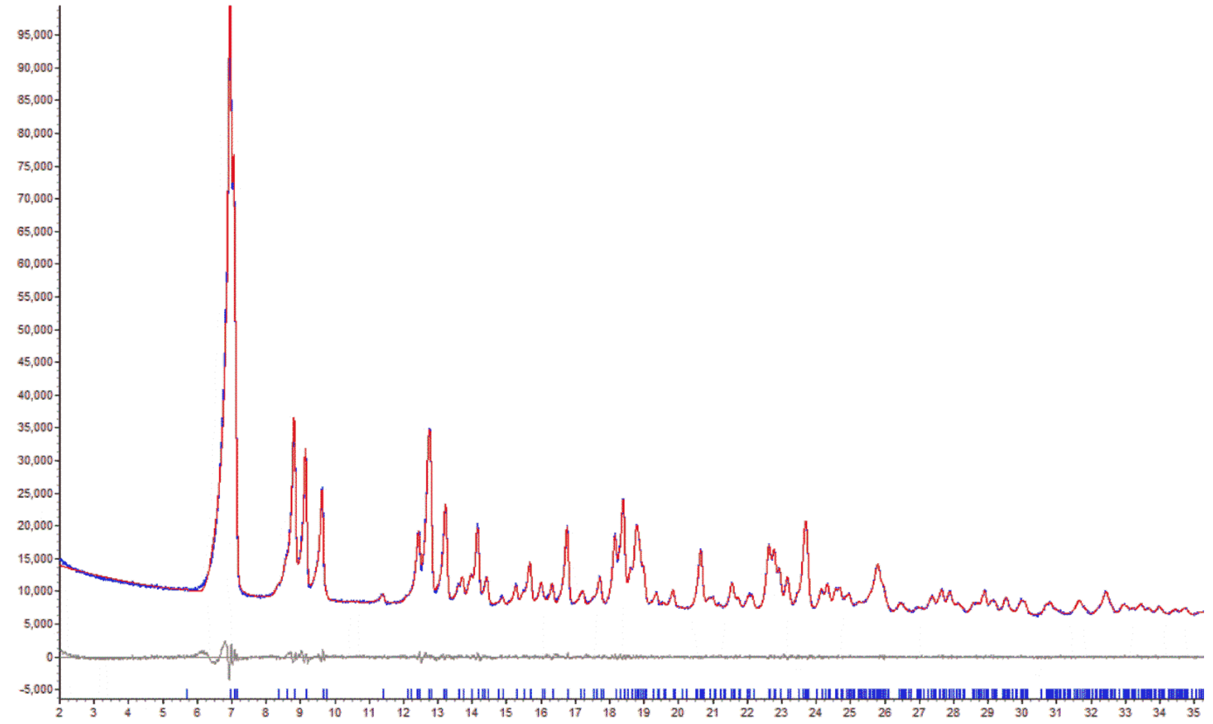

Figure S3. Powder pattern (2θ=2-35°) of the solid after 1 day of studies. Experimental (blue), calculated (red) and differential (gray) lines correspond to the following unit cell parameters: a = 14.4324(9)Å, b = 14.6838(11)Å, c = 15.6863(10)Å, α = 83.572(2)°, β= 99.078(3)°, γ = 120.365(3)°, V= 2830.3(3) $Å^3$. Systematic absences are consistent with the space group $P\bar{1}$.

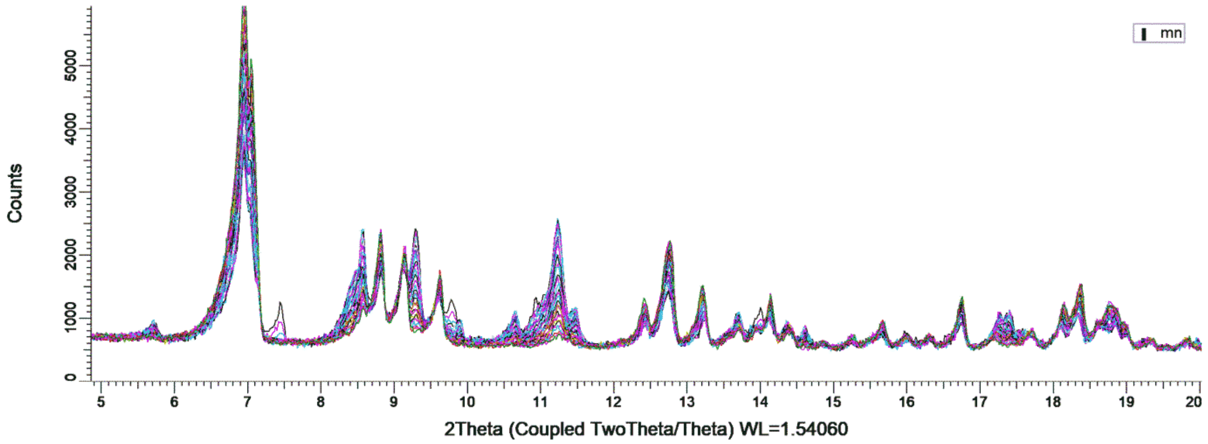

Figure S4. Change in the phase composition of the sample within the first day (2θ=5-20°).

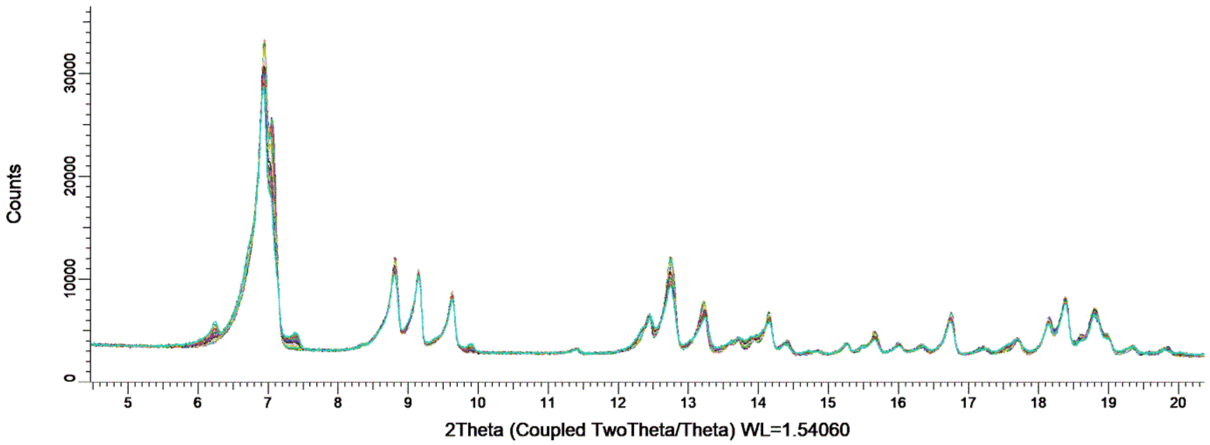

Figure S5. Change in the phase composition of the sample within the 2nd-4th days (2θ=5-20°).

References

Bruker (2008). *APEX2*. Bruker AXS Inc.,Madison, Wisconsin, USA.

Bruker (2015). *TOPAS*. Version 5.0. Bruker AXS, Karlsruhe, Germany.

Sheldrick, G. M. (2008). *Acta Cryst.* **A64**, 112-122.

Sheldrick, G. M. (2015). *Acta Cryst.* **C71**, 3-8.
